# Quantitative determination of niraparib and olaparib tumor distribution by mass spectrometry imaging

**DOI:** 10.7150/ijbs.41395

**Published:** 2020-02-21

**Authors:** Lavinia Morosi, Cristina Matteo, Tommaso Ceruti, Silvia Giordano, Marianna Ponzo, Roberta Frapolli, Massimo Zucchetti, Enrico Davoli, Maurizio D'Incalci, Paolo Ubezio

**Affiliations:** 1 Istituto di Ricerche Farmacologiche Mario Negri IRCCS, Department of Oncology; 2 Istituto di Ricerche Farmacologiche Mario Negri IRCCS, Laboratory of Mass Spectrometry

**Keywords:** drug distribution, mass spectrometry imaging, PARPi

## Abstract

**Rationale**: Optimal intratumor distribution of an anticancer drug is fundamental to reach an active concentration in neoplastic cells, ensuring the therapeutic effect. Determination of drug concentration in tumor homogenates by LC-MS/MS gives important information about this issue but the spatial information gets lost. Targeted mass spectrometry imaging (MSI) has great potential to visualize drug distribution in the different areas of tumor sections, with good spatial resolution and superior specificity. MSI is rapidly evolving as a quantitative technique to measure the absolute drug concentration in each single pixel.

**Methods**: Different inorganic nanoparticles were tested as matrices to visualize the PARP inhibitors (PARPi) niraparib and olaparib. Normalization by deuterated internal standard and a custom preprocessing pipeline were applied to achieve a reliable single pixel quantification of the two drugs in human ovarian tumors from treated mice.

**Results**: A quantitative method to visualize niraparib and olaparib in tumor tissue of treated mice was set up and validated regarding precision, accuracy, linearity, repeatability and limit of detection. The different tumor penetration of the two drugs was visualized by MSI and confirmed by LC-MS/MS, indicating the homogeneous distribution and higher tumor exposure reached by niraparib compared to olaparib. On the other hand, niraparib distribution was heterogeneous in an ovarian tumor model overexpressing the multidrug resistance protein P-gp, a possible cause of resistance to PARPi.

**Conclusions**: The current work highlights for the first time quantitative distribution of PAPRi in tumor tissue. The different tumor distribution of niraparib and olaparib could have important clinical implications. These data confirm the validity of MSI for spatial quantitative measurement of drug distribution providing fundamental information for pharmacokinetic studies, drug discovery and the study of resistance mechanisms.

## Introduction

Ovarian cancer is a worldwide leading cause of death from gynecologic malignancies with overall survival less than 45% within five years from diagnosis 1. Despite a high initial response rate to platinum (Pt) and taxane treatments, most patients relapse with a median progression‐free survival of 18 months, gradually becoming Pt-resistant 2. Recent clinical trials with Poly (ADP-ribose) polymerase inhibitors (PARPi) have shown great clinical benefit in ovarian cancer patients, with substantial progression‐free survival advantages over placebo in the maintenance setting 3-10. The inhibition of PARP activity in homologous recombination-deficient cells (such as those with *BRCA* mutations) induces 'synthetic lethality' due to the simultaneous loss of both single strand break and homologous recombination-mediated double-strand break repair machineries, causing selective cytotoxicity for tumor cells 11-15.

The first Food and Drug Administration PARPi approved for BRCAmut ovarian cancer was olaparib while niraparib was the first PARPi that demonstrated clinical efficacy in both *BRCA*wt and *BRCA*mut tumors 3. In addition, niraparib broader clinical activity might be intrinsically linked to its biophysical properties, such as better water solubility, higher membrane permeability and greater distribution volume 16. All these factors may contribute to a different pharmacokinetic profile, achieving higher tumor exposure to niraparib than olaparib. This could explain why -particularly in *BRCA*wt models- niraparib was more effective than olaparib *in vivo*, although the two drugs have similar *in vitro* PARP catalytic inhibition potency and cytotoxicity in *BRCA*mut cells 16.

The determination of tumor drug concentrations and distribution is important because in order to be effective, an anticancer drug has to reach an active concentration in cancer cells. Pharmacokinetic analysis, monitoring circulating drug concentrations, are not necessarily predictive of the concentration achieved in the tumor because of its complex and poorly/irregularly vascularized microenvironment 17. Measuring drug concentrations in tumor homogenates by LC-MS/MS adds valuable knowledge about this issue, but completely ignores the heterogeneity of drug distribution, losing the spatial information 18. The irregular drug delivery in tumor tissue is instead a critical problem, mainly due to the altered tumor microenvironment. The abnormal blood and lymphatic vessels, inflammation, dense and fibrous connective tissue characterizing the neoplastic phenotype increase interstitial fluid pressure (IFP), solid stress and hypoxia that limit the distribution and efficacy of anticancer drugs 17,19. Another factor influencing the intracellular concentrations of many drugs is the expression and function of membrane transporters. This is particularly important for anticancer drugs as tumor cells often overexpress the ABC transporters (e.g. P-glycoprotein, P-gp) involved in the extrusion of drugs from the cell, thus causing multidrug resistance 20. Some findings suggest that overexpression of ABC transporters might induce resistance to PARP inhibitors (PARPi) too 21. Olaparib and niraparib in fact were reported to be a substrate for P-gp 22,23.

Only few data are available about PARPi distribution in tumor tissue in the literature. Some imaging results indicated that distribution of olaparib through tumor tissue was quite uniform and uptake into cancer cells is sufficiently high to induce molecular effect 24-26. In contrast, the visualization of rucaparib and veliparib in xenograft models indicated that their intratumor distribution was highly heterogeneous 27,28. To our knowledge, no information has been published on the intratumor distribution of niraparib.

To look into this properly, an accurate method is essential to obtain information on the spatial distribution of the drug in tumor tissue. Matrix-assisted laser desorption ionization (MALDI) mass spectrometry imaging (MSI) has evolved as a valuable tool for the qualitative visualization of drugs in tumor slices, with high spatial and mass resolution and great specificity and without the need of labeling the analytes 29-34. Our group has already reported that the intratumor distribution of small molecules is extremely heterogeneous in different tumor models. Drug distribution seems very low and irregular in solid tumors with large necrotic or fibrotic areas 35,36 and irregular vasculature 37. MSI of pharmaceutical compounds has been performed not only using MALDI ion source but even with two alternative ionization technique: secondary ion mass spectrometry (SIMS) and desorption electrospray ionization (DESI). SIMS can achieve the best spatial resolution (<250nm) but with limited sensitivity while DESI allows rapid image acquisition with almost no need of sample preparation 38. However, the most suitable and widely applied ionization technique for quantitative analysis of exogenous compound in treated animals is MALDI 38. MALDI-MSI is now moving forward to overcome its main limitation: the difficulty of acquiring reliable absolute quantitative measurements of the amount of drug in each pixel of the image 39,40. Quantitative MSI has in fact to deal with several issues: sensitivity is limited by the small sample size (e.g. a single pixel); ion intensity can vary widely because of uneven matrix deposition or differences in extraction efficiency 41; the ionization efficiency is strongly analyte-dependent and influenced by ion suppression effect due to the local composition of the surface 42,43. Drug ions identification is further complicated by their very low relative intensity in the spectrum because of their limited concentration in tissue compared to endogenous molecules, especially when potent anticancer drugs are administered at very low doses. Moreover, the small molecules mass range is dominated by matrix fragment and cluster signals that further mask drug-related ion signals 44. Finally a crucial step is the building of a reliable calibration curve prepared in the most similar conditions possible to the analyzed samples 45.

In this study, we developed a mass spectrometry imaging method to obtain for the first time quantitative information at single pixel level about the distribution of olaparib and niraparib in tumor tissue.

We applied the widely accepted normalization method based on stable isotope added to the matrix to take into account the ion suppression and recovery variability 36,39,46. We also tested different inorganic nanoparticles as a MALDI matrix to ensure the almost complete absence of background signals from matrix degradation in the drug mass range, as well as optimal spatial resolution and less recovery problems by avoiding the co-crystallization process 47,48. The preprocessing steps allow to correctly identify and integrate the low intensity ion peaks and to convert the ion signal intensity into drug concentration units using a calibration curve made with the spot on tissue approach in each MALDI detection plate 49. Moreover, we proposed one of the first attempt of rigorous method validation in the emerging field of quantitative MSI 29,42,50.

The MSI method was applied to determine the intra-tumor distribution of niraparib and olaparib in A2780 ovarian cancer model. Overall tumor drug exposure was consistent with LC-MS/MS quantitative analysis on the second half of the same tumors. Niraparib distribution was also analyzed in A2780 resistant cancer model overexpressing P-gp to investigate the role of this transporter in PARPi accumulation in tumor and efficacy.

## Materials and Methods

### Drugs and reagents

Olaparib (batch #24830 MedChemExpress) and niraparib (batch #BO160P003 MedChemExpress) were provided by TESARO Inc (Waltham, MA, USA). Drugs were dissolved in DMSO. Serial dilutions were prepared in 50% ethanol from 5 to 250 pmol/µL for MSI. For chromatographic analysis, niraparib was dissolved in H_2_O:CH_3_CN, 1:1 (v/v) and diluted to obtain standard and quality control (QC) working solutions at 1, 5, 10, 25, 50 and 100 ng/sample and 2.5, 40 and 80 ng/sample, respectively. Serial dilutions of olaparib in methanol were done to prepare standard working solutions at 0.5, 2.0, 5.0, 10.0 and 50.0 ng/sample and the QC working solutions at 4, 25 and 40 ng/sample.

Niraparib-D7 (batch #TJ1-2017-137T Charles Rivers) was dissolved in DMSO at the concentration of 1mg/mL and olaparib-D8 (batch #ALG-ALS-12-072-P3 Alsachim) was dissolved in methanol at a nominal concentration of 1 mg/mL.

For treatment purposes niraparib was dissolved in 10% DMSO-methyl cellulose 0.5% and olaparib in 10% DMSO-Hydroxypropyl)-beta-cyclodextrin 10%.

Gold nanoparticles (AuNPs, 0.7 mM in 50% ethanol synthesized in-house as described in our previous publication 48; titanium dioxide nanoparticles (TiO_2_NPs, 1 mg/mL in 50% ethanol / 0.5% KCl Evonik Industrials, Essen, Germany) and titanium dioxide conjugated with gold nanoparticles (AuTiO_2_NPs, 0.5% gold w/w dissolved at the concentration of 3 mg/mL in 50% ethanol kindly provided by THETIS S.p.A. and produced as described in the Supplementary Material).

TiO_2_-based NP suspensions were vortexed and sonicated for 3 min just before use, to reduce agglomeration and sedimentation.

### Animals and treatments

Experiments involving animals were conducted in conformity with the following laws, regulations, and policies: Italian Governing Law (D.lgs 26/2014; Authorization n.19/2008-A issued March 6, 2008 by Ministry of Health); Mario Negri Institutional Regulations and Policies providing internal authorization for persons conducting animal experiments (Quality Management System Certificate - UNI EN ISO 9001:2008 - Reg. N° 8576-A); the NIH Guide for the Care and Use of Laboratory Animals (2011 edition) and EU directives and guidelines (EEC Council Directive 2010/63/UE) and in line with guidelines for the welfare and use of animals in cancer research 51. Animal experiments were reviewed and approved by the Mario Negri Animal Care and Use Committee (IACUC), which includes members *ad hoc* for ethical issues. Animals were housed in the Institute's Animal Care Facilities, which meet international standards; they are regularly checked by a certified veterinarian who is responsible for health monitoring, animal welfare supervision, experimental protocols and review of procedures.

Ten million A2780wt or A2780/P-gp cells were injected subcutaneously into the right flank of seven-week-old female CD1 nude mice (Charles River, Calco, Italy). When tumor weight reached approximately 400 mg, mice were treated either with vehicle (10% DMSO-methyl cellulose 0.5% for niraparib and 10% DMSO-Hydroxypropyl)-beta-cyclodextrin 10% for olaparib) or with a single dose of niraparib (50 mg/kg p.o.) or olaparib (67 mg/kg p.o) and euthanized 2h after treatment by exposition to increasing CO_2_ concentration. The doses were chosen to reproduce the clinical treatment. The vehicle treated tumors were used for blank evaluation and scale calibration. Tumors were explanted and divided into two parts: the first one was immediately snap-frozen in liquid nitrogen and stored at -80°C until MSI analysis while the second one was stored at -20°C for LC-MS/MS analysis.

### Matrix selection for MSI

For the initial setup, 1 µL of 100 pmol/µL drug standard solutions dissolved in 50% ethanol were spotted onto the steel MALDI plate (Opti-TOF 384 Well insert). After complete air-drying, the drug spot was covered with 1 µL of different NP suspensions to be tested as matrices.

To assess the influence of biological matrix on drugs ionization, niraparib and olaparib standards were spotted (0.2 µL *per* spot) at increasing concentrations (5, 10, 20, 50 and 100 pmol/spot) on untreated tumor sections. The parameters evaluated to compare the ionization efficiency were the drug signal intensity and the signal to background intensity ratio (calculated as the ratio of the mean signal intensities in the drug spots to the blank spot). The ion signal intensity in the spots over tissue section is compared with the ion signal intensity on MALDI plate to address the ion suppression problem.

Frozen tumors were cut into 10 μm thick sections using a cryo-microtome (Leica Microsystems, Wetzler, Germany) at -20°C and mounted on a pre-cooled MALDI plate (Opti-TOF 384 Well insert) by standard thaw-mounting techniques. The plate was dried in a vacuum drier at room temperature overnight. Then each plate was sprayed with AuNPs, TiO_2_NPs or AuTiO_2_NPs matrix suspensions using a BD 180 precision double-action trigger airbrush (FENGDA, Zhejiang, China) with a 0.20 mm nozzle diameter, using nitrogen at 0.2 atm. Typically 5 mL of matrix solution was sprayed on each 3cm x 3cm portion of MALDI plate.

A MALDI 4800 TOF-TOF (AB SCIEX Old Connecticut Path, Framingham, MA 01701, USA) was used, equipped with a 355 nm Nd:YAG laser with a 200 Hz repetition rate and 10µm spot size, controlled by the 4000 Series ExplorerTM software (AB SCIEX Old Connecticut Path, Framingham, MA 01701, USA). MS were acquired in reflectron with 20 laser shots with intensity of 4500 arbitrary units, with a bin size of 0.5 ns. The instrument was mass calibrated with a CAL-MIX before analysis and used at 10,000 resolving power.

Images of tissue sections were acquired using the 4800 Imaging Tool software (www.maldi-msi.org, M. Stoeckli, Novartis Pharma, Basel, Switzerland), with an imaging raster of 100x100 µm.

Tissue View software 1.1 (AB SCIEX) was used to process and display the ion distribution inside the tumor sections in this preliminary setup phase. Niraparib and olaparib were imaged by plotting K^+^ or Na^+^ adduct ions at m/z 359.1 or m/z 343.1 and m/z 473.1 or m/z 457.1 respectively. A classical RAINBOW color scale was used to create images of drugs, setting the saturation following the maximum in each acquisition.

### Quantitative MSI

D7-niraparib or D8-olaparib 3 μg/mL were added to the matrix as internal standard (IS) before spraying and uniformly applied over tissue sections. The IS ion signals has a double function in each pixel: to find the m/z range where the drug ion signal should be identified and then to normalize the signal intensity. This procedure is essential to correct the resulting images in order to take into account the laser shot-to-shot variability and the different ion suppression and/or experimental m/z drifts during acquisition due to the tissue heterogeneity 29.

Molecular images were generated and analyzed using a custom data processing pipeline 49 adapted for niraparib and olaparib. The method (described in details in the Supplementary Material) comprised several steps: data extraction from raw MS acquisition, identification of the m/z position of the peaks of interest, integration of the signals over (pixel-specific) optimized m/z ranges, tissue identification, normalization with the internal standard and noise reduction applying a median filter (3x3) to the 2D image matrix. Then 2D images of the normalized drug-related signals were converted in “quantitative” images by applying the parameters of the calibration curve. In this work, the calibration curve was built by spotting increasing standard drug concentrations (1, 2, 5, 10, 20 and 50 pmol/spot) on a section of untreated tumor in each MALDI plate. We considered a ROI within each spot (including typically 100 pixels and excluding the edges) and we assumed the signals of pixels within a ROI as replicate measures of the same nominal drug concentration 50. The mean normalized ion signal and the standard error (SE) inside these ROIs were plotted against the respective nominal concentrations to build the calibration curve, obtained with a weighted linear fit with 1/SE^2^ weight. The weighting allows compensation over the different variances of the mean signal intensities within the spots at different concentrations. The nominal concentration (pmol/mm^2^) of each ROI was obtained dividing the overall drug amount spotted, by the exact number of pixels in the ROI (converted to mm^2^ taking into account the dimension of each pixel, 100μm x 100μm).

**Figure 1:**
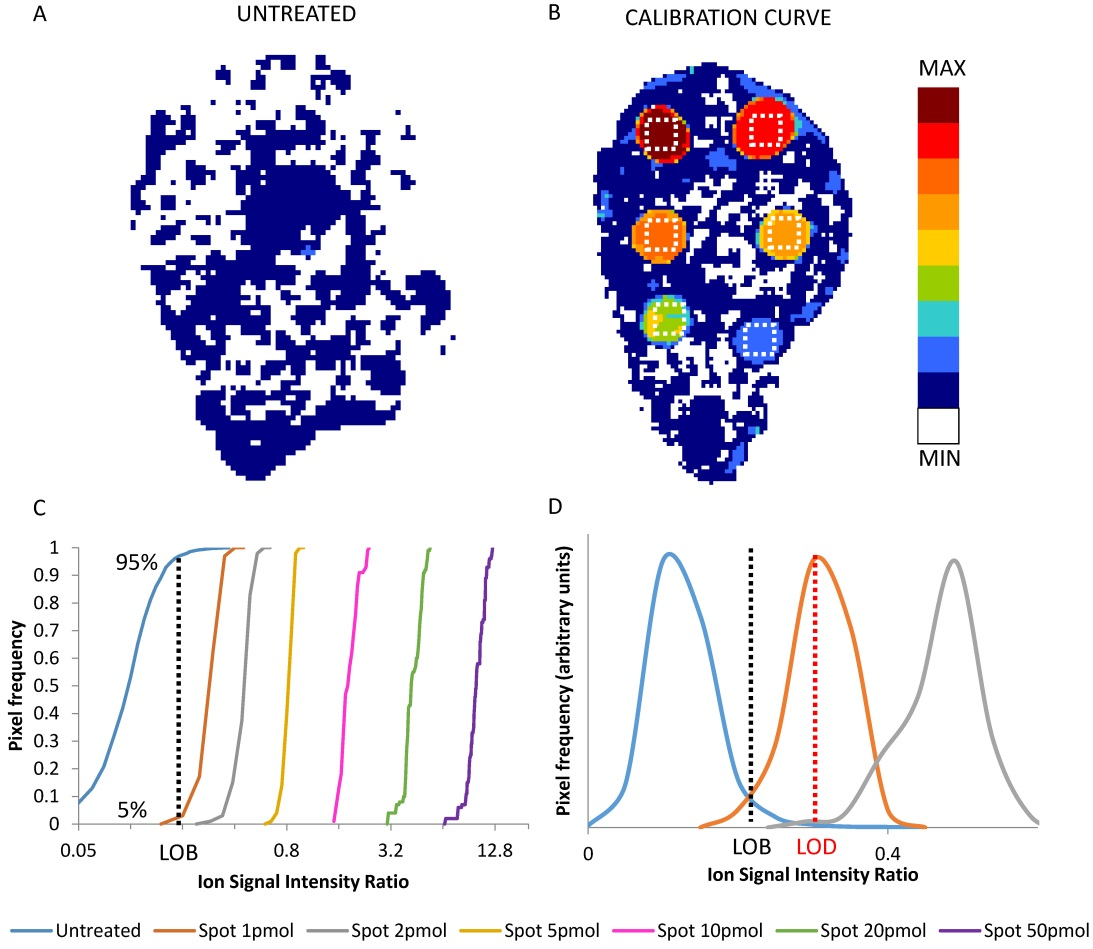
2D images of the normalized niraparib ion signal after application of the median filter (3x3) in an untreated tumor (A) and in a calibration curve section (B). The dotted white square depict the 100 pixel ROIs taken into account for calibration. Cumulative distribution of normalized drug-related signals in the pixel of untreated tumor and of the ROIs of each calibration spot (C). The value at 95% of the distribution of untreated tumor (dotted black line) corresponds to the LOB. The LOD correspond to the mean of first distribution whose only the 5% is under the LOB (D). In this example the LOD is the mean of the signals of spot 1pmol (dotted red line).

The frequency distributions of the replicated signals in each ROI and in an untreated tumor section (blank) were used to state the limit of blank (LOB) and limit of detection (LOD) (Figure 1) 52. LOB was set as the signal level not exceeded by 95% of the pixels in untreated control sections. LOD was taken as the average signal in the lowest nominal drug concentration ROI of the calibration scale, wherein only the 5% of the pixels were under the LOB 45,53. The parameters of the calibration curve were used to transform the 2D images of the normalized drug-related signals into quantitative images of the spatial distribution of the drug concentration in each pixel (pg/pixel). The corresponding concentration per grams of tissue (μg/g) was estimated considering the depth of the section (10 μm) and assuming unitary density.

We included the LOD information in the color scale of the final quantitative images of the drug distribution, painting dark blue the pixels with drug signals below the LOD, above the average of the blank, and white those pixels with signals even below the average of the blank.

The method was validated by adapting for MSI, the generally accepted guidelines for HPLC bioanalytical methods 53-55. We assessed the following parameters for niraparib and olaparib imaging: precision, goodness of the fitting, repeatability, limit of detection and accuracy.

The precision of the method was determined using the CV% at two different levels:

1) the pixel level (0.01 mm^2^_,_ rougly 0.1 μg tissue) comparing the pixels in the ROIs within each calibration spot.

2) the ROI level (1 mm^2^, roughly 0.01 mg tissue) comparing average concentrations in replicate spots.

Under these experimental conditions, the goal was mean precision determined at each level not exceeding 20% of the CV%.

The accuracy was expressed as the percentage deviation between the mean calculated concentrations and the nominal concentration at ROI level. We consider acceptable mean values within 20% of the nominal value (25% for the lowest calibration point).

Three calibration curves of niraparib and olaparib were analyzed on the same working day including six spots to determine the precision at single-pixel level, to assess the goodness of the fitting and the intra-day repeatability.

The goodness of weighted fits was evaluated by the standard deviation (SD) and the CV% of best fit slope (m) and y intercept (q) and the plot of the standardized residuals, calculated as the ratio of the percentage residuals (the difference between the measured Y value and the Y value predicted by the calibration equation) to the standard deviation of the measures.

The intra-day repeatability was assessed comparing m and q of the three calibration curves considering acceptable an inter-curve CV% of these parameters lower than 25%.

The precision (at ROI level) and accuracy were checked by measuring the analytes in three replicates of QC samples spotted on untreated tumor sections. Two QC levels were analyzed at the concentrations of 3 and 7 pmol/spot. Moreover, the inter-day repeatability of the calibration scales was validated over three working days together with the accuracy of the back-calculated concentrations.

The analytical method was applied to a preliminary drug distribution study in ovarian cancer models (A2780wt or A2780/P-gp). For niraparib and olaparib drug distribution experiments, four tumors per group and three sections per each tumor were analyzed. One 10 μm section every 300 μm of tumor tissue was cut and mounted on MALDI plate, the adjacent one was mounted on a glass slide and stored at -20°C for H&E staining. The percentage of pixels where the drug is over the LOD has been calculated as a parameter to compare the extent of drug diffusion in the analyzed sections.

### Quantitative LC-MS/MS analysis

On the second half of each tumor analyzed by MSI we measured the total drug concentration in tissue homogenate by LC-MS/MS.

For niraparib in tumor tissues the method was adapted from Sun et al. 16. Briefly, tumors were homogenized 1 g: 10 mL (w/v) in NH_4_CH_3_COOH 20 mM and 100 µL of unknown samples were transferred to polypropylene tubes to be extracted. Six-point calibration curve and three replicates of each QC concentration were prepared spiking 5 µL of the corresponding working solution into 95 µL of control matrix homogenate. To all samples analyzed we added 5 µL of IS (D7-niraparib) at the final concertation of 25 ng/sample (2.75 µg/g), vortexed and extracted with 0.5 mL of CH_3_CN:CH_3_OH solution 1:1 (v/v). Samples were centrifuged for 10 min at 13200 rpm 4°C and the supernatant was dried under N_2_ at 40°C and reconstituted in 120 µL of a MP-A:MP-B 1:1 (v/v) solution (MP-A: CH_3_COONH_4_ 20 mM; MP-B: CH_3_OH:CH_3_CN 1:1 (v/v), HCOOH 0.1%). After vigorous vortex mixing, samples were centrifuged again at 13200 rpm for 10 minutes at 20°C, then the supernatant was transferred to a glass vial and 2 µL were injected into the HPLC-MS/MS system.

For olaparib quantification in tissues, the method was adapted from Nijenhuis et al. 56. Tumors were homogenized 1 g: 10 mL (w/v) in CH_3_COONH_4_ 20 mM and 25 μL of tumor homogenate was further diluted with 10%BSA, 1:1 (v/v). D8-olaparib (50 ng) dissolved in methanol was added to all the samples. For extraction 1 mL of methyl tert-butyl ether was added with shaking for 5 min and centrifuging at 13000 rpm for 10 min. The samples were snap-frozen and the organic layer was properly separated and removed. Extracted samples were evaporated under nitrogen, then reconstituted in 100 μL of methanol, and 2 μL were injected into the HPLC-MS/MS system for quantification.

MS detection was carried out on a triple quadrupole API 4000 mass spectrometer (Sciex, MA, USA) equipped with electrospray ionization (ESI) operating in positive ion mode.

The chromatographic separation of both drugs was obtained with a Gemini C18 column, 50 mm×2·0 mm, 5 μm (Phenomenex, Torrance, USA) at 40°C, protected with Security Guard™ ULTRA cartridges C18 (Phenomenex Inc., Torrance, CA, USA).

For quantification we monitored the transitions 435.40>367.10 m/z. and 321.0>304.1 of olaparib and niraparib, respectively.

### Statistical analysis

Fitting of the calibration scale and statistical analyses were performed with GraphPad Prism version 6.01 software (GraphPad software, Inc., La Jolla, CA, U.S.A.). Shapiro-Wilk normality test and runs-test were used to analyze residuals to test the goodness of the fitting 57,58. Student's t test was performed to evaluate differences in drug distribution experiments.

## Results and Discussion

### Matrix selection for MSI

The mass spectra obtained with three different matrices are shown in the Supplementary Material (Figures S1-S6). The ionization best was obtained for both drugs with AuTiO_2_NPs as Na^+^ or K^+^ adducts (for niraparib m/z 343.1 and m/z 359.1 and for olaparib m/z 457.1 and m/z 473.1 respectively) in positive ion mode, as described in detail in the Supplementary Material.

Table 1 summarizes the main ion signals intensity of niraparib and olaparib (Na^+^ or K^+^ adduct depending on the drug/matrix combination), spotted at increasing concentrations on untreated tumor sections. The lowest detectable niraparib concentration on tissue with AuNPs and TiO_2_NPs matrices was that of the 10 pmol spot, while the 5 pmol spot was well detectable only with AuTiO_2_NPs. Olaparib was detectable in the 20 pmol spot with AuNPs, only in the 50 pmol spot with TiO_2_NPs (with negative ionization), while AuTiO_2_NPs again performed better, enabling the detection of the 5 pmol spot. AuTiO_2_ gave the best results not only in terms of signal intensity but even evaluating background intensity to signal ratio (Table 1).

As regard the effect of biological matrix on ionization, a 100 fold decrease in niraparib signal intensity resulted on tissue compared to MALDI plate with AuNPs and TiO_2_NPs while the ion suppression was less marked with AuTiO_2_NPs (40 fold). For olaparib, ion suppression caused a 20 time decrease in ion signal intensity on tissue compared to MALDI plate with AuNPs and TiO_2_NPs while only a 4 time decrease with AuTiO_2_NPs.

In conclusion, the most suitable matrix for imaging experiments was AuTiO_2_ in positive ion mode, since it allowed visualization of the lowest drug concentration with a very low background noise.

### Quantitative MSI method validation

LOD determination is an often neglected but vital step to ensure correct interpretation of MSI quantitative data, especially when the relationship between tissue drug distribution and the efficacy of therapy is under investigation 50. A formal assessment of the LOD requires taking account not only of the variability of the blank, but also the precision of the detection within the standard spots, here in ROIs where variability of the actual drug concentration can be neglected. Figure 1 panel A and B shows an example of an untreated tumor section and of a calibration curve section respectively, while panel C shows the frequency distributions of normalized drug-related signals in the ROIs of the spots at increasing drug amount. The average normalized drug-related signal of the frequency distribution which had the 5th percentile equal to the LOB (95^th^ percentile of blank) was used to calculate the LOD. Then the average normalized drug-related signal of each ROI with the respective nominal concentration were used to build a calibration curve.

The LOD was found near to the 1 pmol spot, corresponding for niraparib a to 1.47± 0.15 pg/pixel, roughly 14.7 ± 1.5 µg/g (mean and SD of the three independent determinations) and for olaparib to 2.24± 0.43 pg/pixel, roughly 22.4 ± 4.3 µg/g.

**Figure 2:**
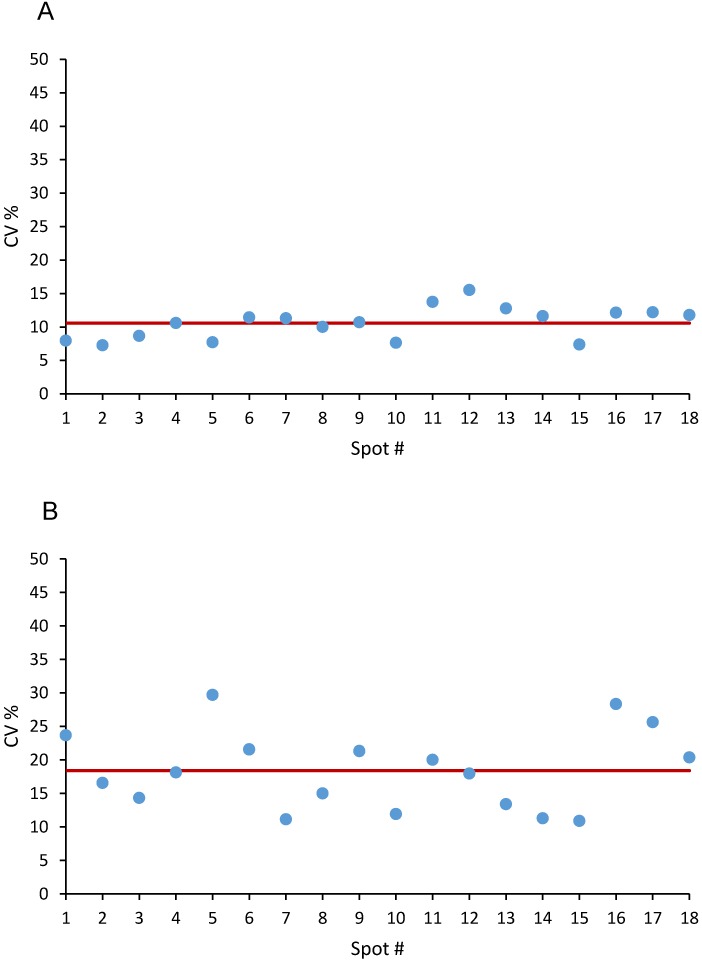
Precision at single pixel level expressed as CV% in 18 spot of Niraparib (A) and of Olaparib (B). The red line corresponds to the mean CV%.

The precision at pixel level was evaluated on the three calibration curves (6 concentrations, 18 spots) spotted on untreated tumor tissue the same working day (Figure 2), The mean CV% for the niraparib ion signal was 10.6±2.4%, (range 7.3-15.5%) (Figure 2A). The mean CV% in the 18 spots for olaparib was higher: 18.4±5.9% (range 11.1-29.7%) (Figure 2B). Considering the complexity of the measure and the small quantity of tissue in each pixel, this level of precision is in our opinion quite acceptable for the aim of construction a quantitative 2D image. Moreover, because the precision of the 1 pmol spots, where the LOD is calculated, was not higher and respected the same acceptability criteria, the limit of quantification was set equal to the LOD.

**Figure 3:**
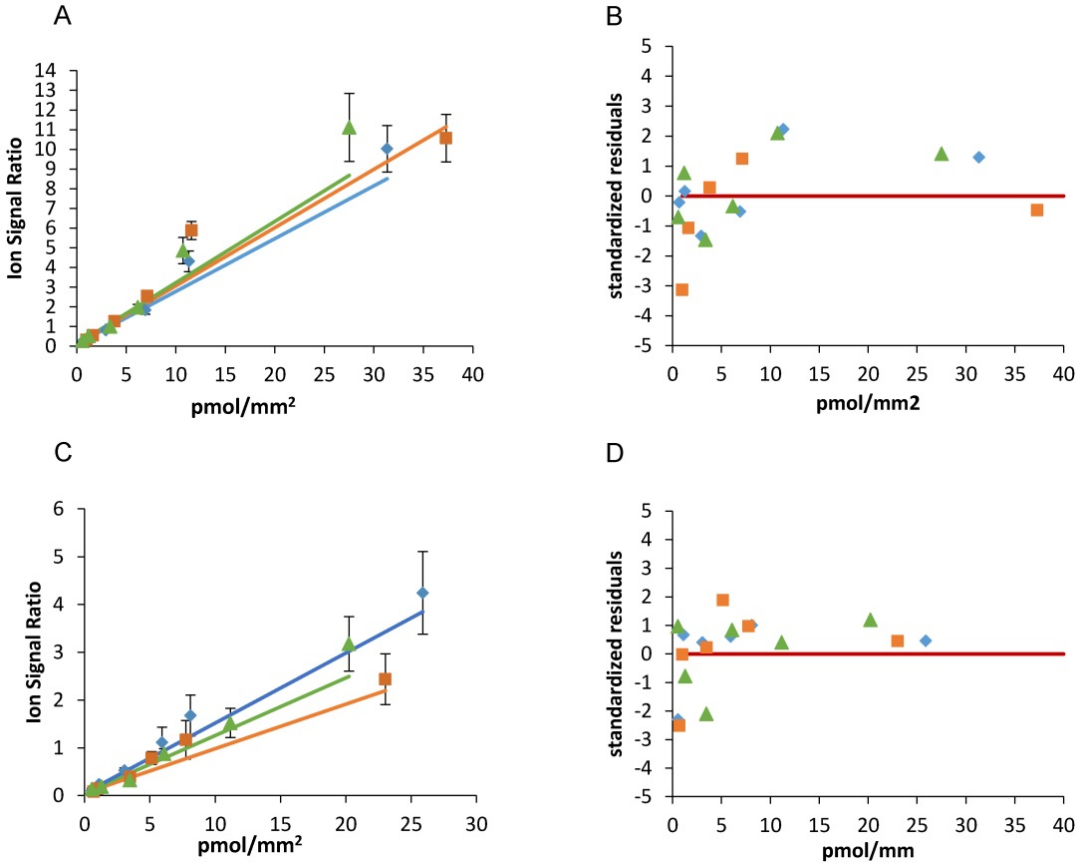
Niraparib (A) and olaparib (C) calibration curves spotted the same working day and the corresponding standardized residuals (B and D). The red lines indicate the zero.

Figure 3 and table 2 show the results of the weighted linear fitting and the intra-day repeatability of the two drugs calibration scales. The slope (m) and intercept (q) were estimated in each curve with CV% <15% and standardized residuals were distributed above and below the zero, satisfying the Shapiro-Wilk normality test with a single exception (panel C blue line). Because systematic deviations from linearity were not observed and were never detected by runs- test, we did not consider more complex (e.g. polynomial) models nor a restriction of the linearity range.

The best fit slope (m) and y intercept (q) of three independent scales of niraparib spotted the same working day were close to each other (m mean±SD: 0.292±0.022, CV%=7.6); q mean±SD: 0.103±0.002, CV%=1.7) confirming the intra-day repeatability.

As regard olaparib, the slope (m mean±SD: 0.120±0.027, CV%=22.3) and y intercept (q mean±SD: 0.053±0.0001, CV%=0.2) were similar comparing the three curves, although with somewhat more variability than niraparib.

For niraparib the concentration accuracy of QCs spotted at 3 and 7 pmol/spot expressed as the percentage deviation was 12.6-17.7% and the precision at ROI level fell into the range 12.4-15.7% (Table 3).

For olaparib the concentration accuracy of QCs expressed as the percentage deviation was 12.2-27.2% and the precision at ROI level fell into in the range 3.4-14.5% (Table 4). For both drugs the precision at ROI level was adequate for all the concentrations and the accuracy was acceptable for an imaging method considering that the percentage deviation from the nominal value of the QCs was less than 20% for at least 67% of the QCs and for 50% of the concentrations. Moreover, the total mean accuracy of the QCs was 19.7% (Table 4).

The inter-day repeatability and the back-calculated concentration accuracy of niraparib and olaparib methods are reported in supplementary information (Figure S8 and Table S1-S2).

It is important to point out the high variability (especially for olaparib) of the best value of the y intercept in different days. This is mainly due to the different background noise from plate to plate. In this situation, it is mandatory to prepare a fresh calibration curve for each plate analyzed in order to determine the specific LOB, LOD, m and q.

### PARPi tumor distribution

The tumor distribution of niraparib and olaparib was analyzed in four tumors per group in mice bearing the A2780*wt* ovarian cancer treated at comparable doses (Niraparib 50 mg/kg p.o.; Olaparib 67 mg/kg p.o.).

**Figure 4:**
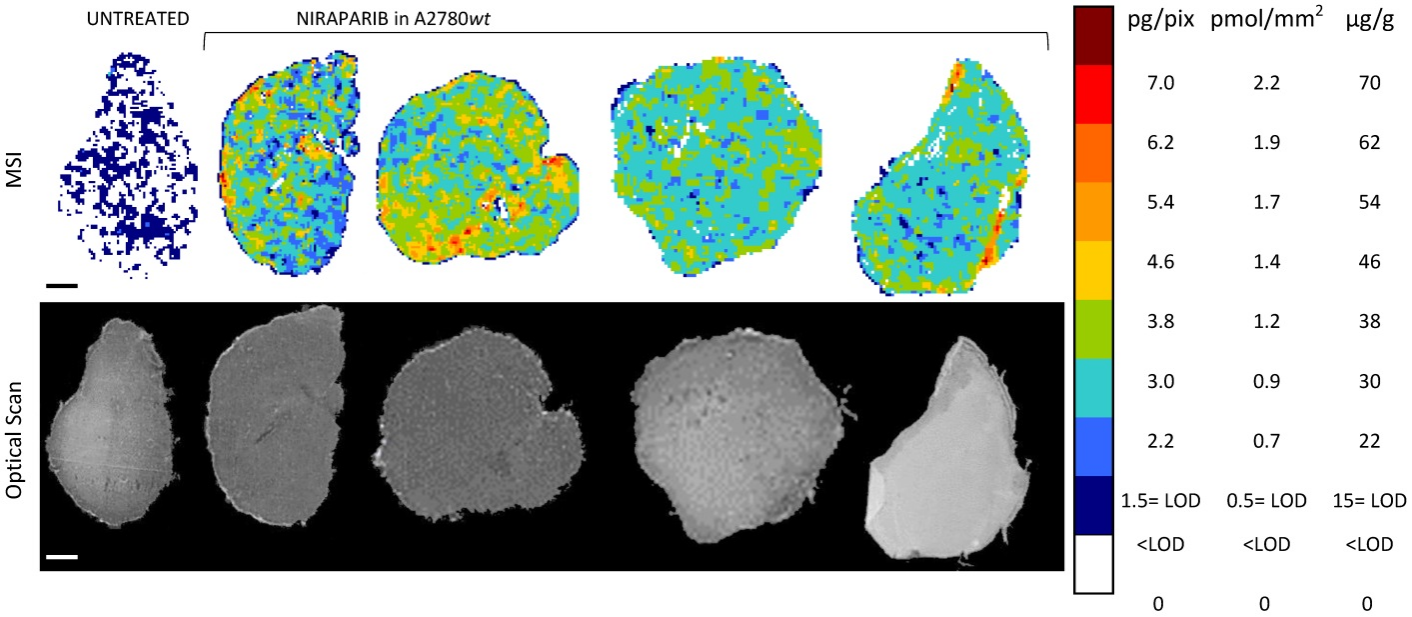
Niraparib quantitative distribution by MSI in the ovarian cancer model A2780*wt*. One representative section of the three analyzed for each tumor is shown. The lower panel shows the corresponding optical scan of the sections. Scale bar: 1mm

**Figure 5:**
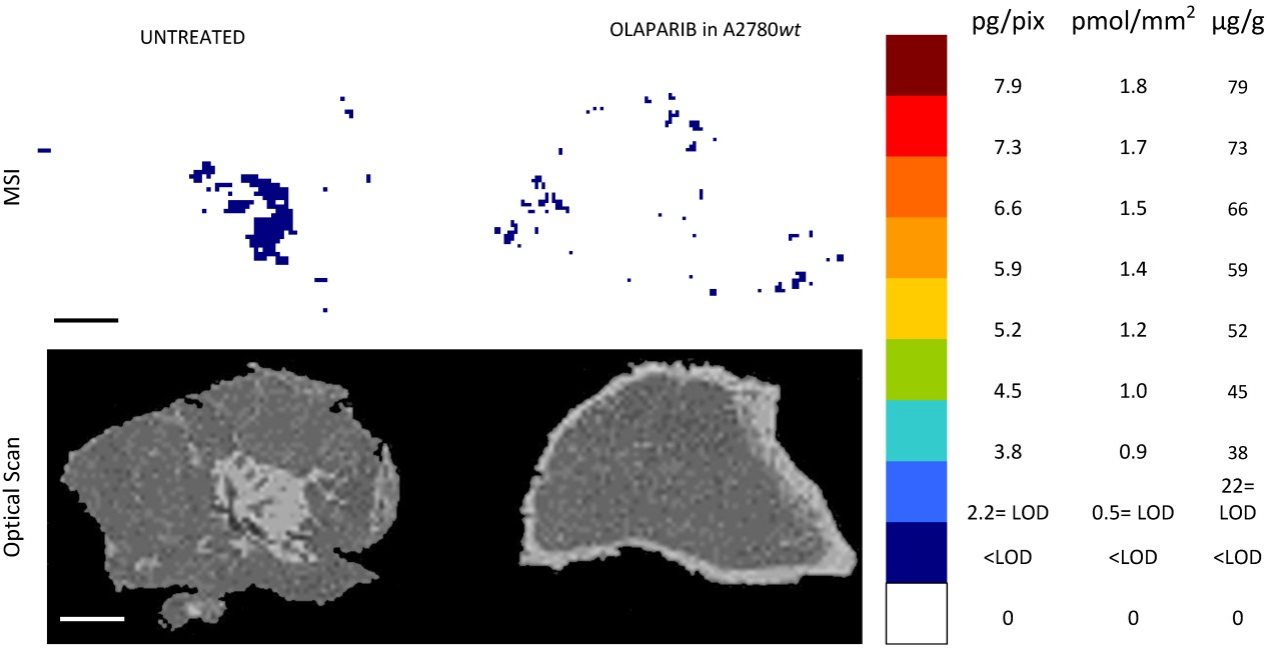
Olaparib resulted undetectable by MSI in the ovarian cancer model A2780*wt*. One representative tumor of the four analyzed is shown. The lower panel shows the corresponding optical scan of the sections. Scale bar: 1mm

The MSI normalized images (Figure 4) show a homogeneous niraparib distribution in A2780*wt*, with low variability within slices of the same tumors and between tumors. The mean CV% of the drug concentration in each pixel was in fact 32.3% (ranging from 24.5% to 42.4%). The olaparib ion signal instead was below the LOD (2.24± 0.43 pg/pixel) in all the sections analyzed, with no appreciable difference from untreated tumors (Figure 5). The different intratumor drug concentration reached by the two drug was confirmed by the parallel LC-MS/MS analysis: the average olaparib concentration was 1.18±0.31 µg/g, 10 times lower than the concentration reached by niraparib (10.40±1.69 µg/g; t test p-value= 0.000025). These data indicate a widely different tumor drug exposure of the two PARPi, administered at comparable doses, as used in clinical practice.

**Figure 6:**
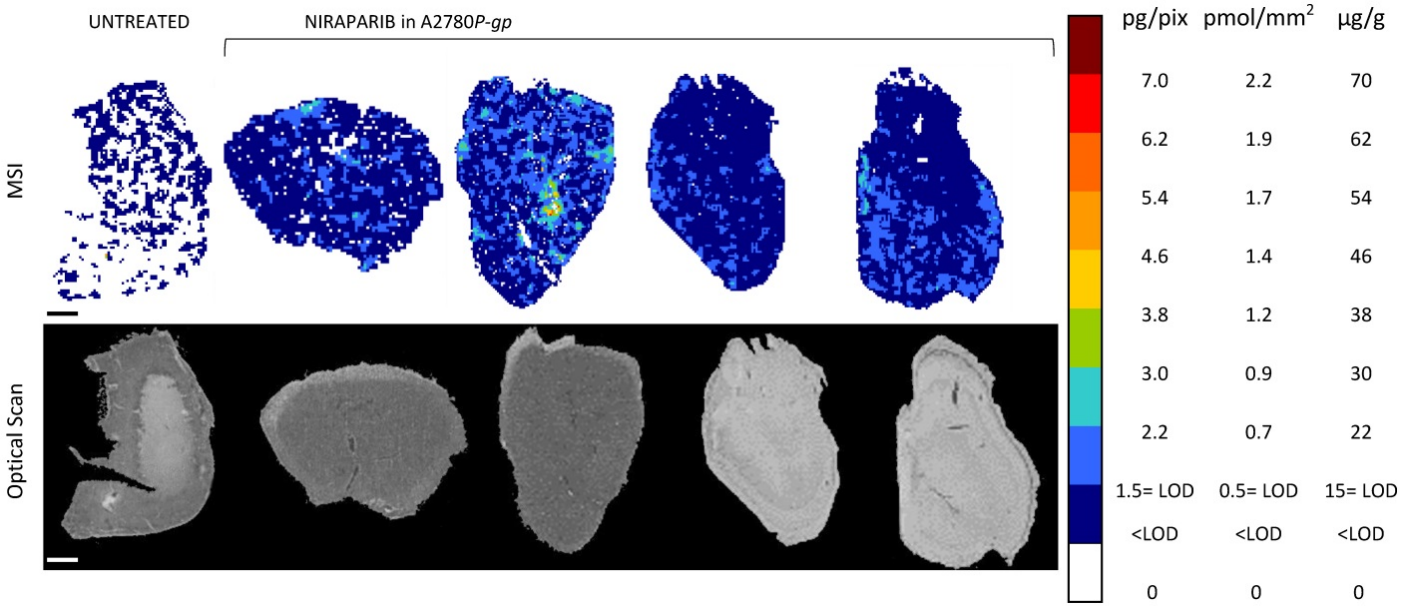
Niraparib quantitative distribution by MSI in the ovarian cancer model A2780*P-gp*. One representative section of the three analyzed for each tumor is shown. The lower panel shows the corresponding optical scan of the sections. Scale bar: 1mm

**Figure 7:**
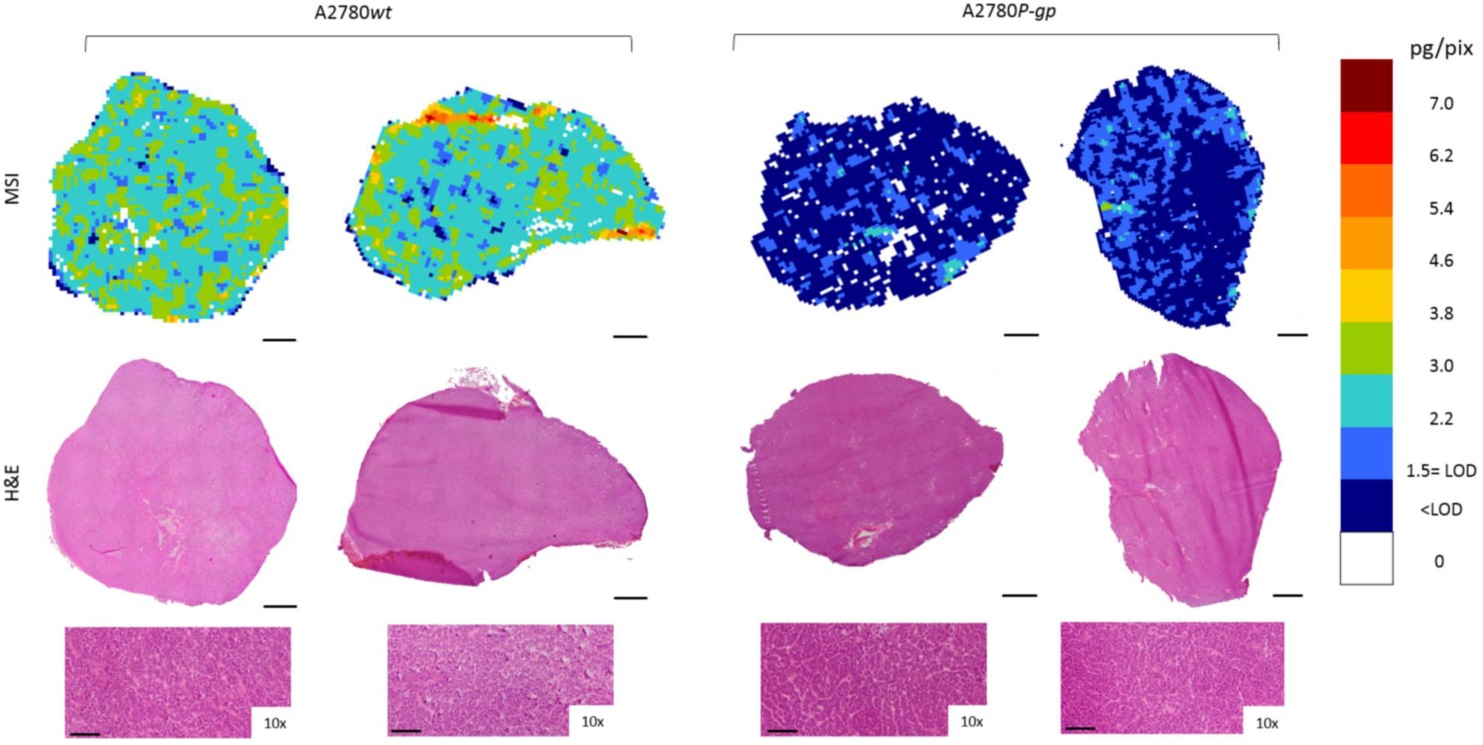
Niraparib quantitative distribution by MSI in the ovarian cancer models compared to H&E staining on the adjacent section. The lower panels show a 10x enlargement of the section. One representative section of the three analyzed for each tumor is shown. Scale bar: 1mm or 100µm (10x images)

Moreover, to investigate niraparib affinity for P-gp, we performed MSI and LC-MS/MS analysis in a resistant ovarian model overexpressing P-gp (A2780*P-gp*). The drug penetration in the *P-gp* model was lower than in *wt* tumors and niraparib reached concentrations above the LOD only in limited tumor areas. In fact the percentage of pixels “positive” to the drug (where the drug concentration is above the LOD) was lower in the *P-gp-*overexpressing tumor model (18.4±8.8%) than its wild type counterpart (87.7±7.4%; p-value test t=0.0000916) in which the distribution homogeneously exceeded the LOD (Figure 6). H&E staining was performed on the section adjacent to the one analyzed by MSI (Figure 7). No macroscopic difference in tissue morphology can be pointed out comparing A2780*wt* and A2780/P-gp or among the area with different drug concentrations. Quantitative LC-MS/MS analysis of tumor homogenates confirmed this different distribution: average tumor concentration of niraparib was double in the wild type than in A2780/P-gp tumors (mean±SD: 10.40±1.69 µg/g in A2780*wt* and 5.29±0.55 µg/g in A2780/P-gp; t test p-value=0.00237). Similarly, the mean drug concentrations calculated locally in the sections by MSI were respectively 25.74±3.20 µg/g and 9.96±2.93 µg/g in A2780*wt* and A2780/P-gp.

Taking account of the diversity of the methods and their limitations, the correlation between LC-MS/ MS and MSI quantitative results was satisfactory (R=0.93, p-value=0.0025) (figure S10 and Table S3).

The differences between the two techniques can be due to several factors. First of all the sampling in different parts of the tumor makes the results not exactly comparable: LC-MS/MS in fact addresses the drug quantification in the whole tumor homogenate while MSI deals with a few sections of the tumor where the distribution can vary widely. In addition, LC-MS/MS measures of drug concentration rely on an accurate measure of the weight of tumor specimen to be analysed, while MSI properly measures a surface concentration in dried microtome-cut sections, which can be converted to μg/g only with assumptions on the section width and specific weight. Moreover, MSI quantification can be slightly influenced by the different extraction of the analyte from the treated samples (from inside the tissue) compared to the calibration curve where the drug is spotted over the tissue. Alternative methods to build a calibration curve taking this problem into account has been published (e.g. mimetic tissue model 41,45), but are extremely time-consuming and not straightforwardly applicable in our experimental conditions because of massive ion suppression in tumor homogenates (data not shown). Therefore, knowing its limitations, the calibration curve spotted on tissue can be considered a reasonable alternative 42. Finally, the higher LOD of MSI than LC-MS/MS could cause the loss of some quantitative information.

## Conclusions

An accurate, reproducible imaging method to determine niraparib and olaparib distribution in tumor tissue is proposed for the first time, allowing the quantification of the two drugs in each pixel of the tissue image, preserving the spatial information. Analysis of niraparib distribution in *wt* A2780 ovarian cancer showed homogeneous penetration inside tumor tissue, while the olaparib ion signal was below the LOD in all the sections analyzed and in fact, no differences could be seen between treated and untreated tumors in molecular images and in the tissue mean spectrum. MSI data were consistent with the measurement of the two PARPi in tumor homogenates by LC-MS/MS. This highlighted an interesting difference in tumor concentration of the drugs administered at therapeutic and comparable doses: niraparib mean concentrations in tumor A2780*wt* were in fact, ten times higher than olaparib. The different concentrations in tumor tissue can partially explain why niraparib, and not olaparib, is active in BRCA*wt* patients 3 thanks probably to the higher drug concentration at the site of action. One can speculate that BRCA-mutated tumors are so sensitive to PARPi that even low concentrations of either olaparib or niraparib induce an antitumor response, whereas antitumor activity against BRCA*wt* tumors requires an higher drug concentration and therefore niraparib has a better chance of being effective. Thus the different pharmacokinetic behavior can partially explain the broader clinical activity of niraparib. The high tumor concentrations of niraparib and longer half-life (36 vs 15 h) suggest that a partial dose reduction should not lead to any loss of drug activity.

Due to the low tumor penetration of olaparib, the MSI method was not sensitive enough to detect the drug in mice treated with a standard schedule. Thus, while the niraparib MSI method was successfully established at a quantitative level, olaparib need further improvements to lower the LOD and overcome the standard criteria for a full validation.

Moreover, the distribution of niraparib was analyzed in a resistant P-gp overexpressing ovarian cancer model, where the drug penetrated the tumor to a lower extent and it was detectable only in small areas of the tumor section. LC-MS/MS determination of drug concentrations in tumor homogenates validated this different distribution. The overexpresssion of P-gp might therefore contribute to the resistance to PARPi.

This study demonstrates the potential of quantitative MSI combined with LC-MS/MS for fuller understanding of anticancer drug intratumor distribution.

We plan to use this method to investigate a broad range of tumors, sensitive and resistant to PARPi to understand whether at least in some cases, e.g. in BRCA*wt* the sensitivity to these drugs is related to the tumor drug concentrations.

## Supplementary Material

Supplementary figures and tables.

## Figures and Tables

**Table 1:** Mean ion signal intensity and the signal to background Ratio ratio for each drug; ND= not detectable

		Mean ion signal intensity			Signal to Background Ratio		
	pmol/ spot	AuNPs	TiO2 NPs	AuTiO2 NPs	AuNPs	TiO2 NPs	AuTiO2 NPs
**Niraparib**	**100**	277.5	149.5	1319.4	15.3	20.8	47.0
	**50**	219.1	78.7	763.1	12.1	10.9	27.2
	**20**	86.9	66.7	445.1	4.8	9.3	15.8
	**10**	61.1	27.6	269.7	3.4	3.8	9.6
	**5**	ND	ND	155.3	ND	ND	5.5
	**Blank**	18.1	7.2	28.1			
**Olaparib**	**100**	745.2	613.5	4701.6	32.7	2.4	230.5
	**50**	321.7	353.4	2490.1	14.1	1.4	122.1
	**20**	176.2	ND	673.1	7.7	ND	33.0
	**10**	ND	ND	271.4	ND	ND	13.3
	**5**	ND	ND	124.6	ND	ND	6.1
	**Blank**	22.8	253.2	20.4			

**Table 2:** Slope and y intercepts values of three calibration curve of niraparib and olaparib spotted in the same working day. SD and CV% is presented for each m and q and comparing different curves.

	Niraparib		Olaparib	
	m±SD (CV%) [U_drug_/(pmol/mm^2^)]*	q±SD (CV%) [U_drug_)]*	m±SD (CV%) [U_drug_/(pmol/mm^2^)]*	q±SD (CV%) [U_drug_)]*
Rep 1	0.296±0.035 (11.8)	0.101±0.016 (15.9)	0.093±0.014 (14.7)	0.0527±0.0014 (2.7)
Rep 2	0.312±0.022 (7.1)	0.104±0.009 (8.5)	0.121±0.012 (9.7)	0.0529±0.0012 (2.3)
Rep 3	0.268±0.018 (6.8)	0.104±0.008 (8.1)	0.147±0.014 (9.4)	0.0528±0.0012 (2.2)
**MEAN**	**0.292**	**0.103**	**0.120**	**0.0528**
**SD**	**0.022**	**0.002**	**0.027**	**0.0001**
**CV%**	**7.6**	**1.7**	**22.3**	**0.2**

* U_drug_ : unit of the normalised drug signal (dimensionless)

**Table 3:** Accuracy and precision at ROI level on QCs at two concentrations of niraparib

Spotted pmol/spot	Nominal pmol/mm^2^	Measured pmol/mm^2^	MEAN	SD	CV (%)	Accuracy (%)	Accuracy MEAN (%)
**7**	5.2	5.1	5.9	0.9	**12.4**	1.7	**17.7**
	4.6	5.7				22.7	
	5.4	6.9				28.8	
**3**	2.1	1.7	1.8	0.2	**15.7**	18.8	**12.6**
	1.9	2.0				4.0	
	1.9	1.6				15.1	

**Table 4:** Accuracy and precision at ROI level on QCs at two concentrations of olaparib

Spotted pmol/spot	Nominal pmol/mm^2^	Measured pmol/mm^2^	MEAN	SD	CV (%)	Accuracy (%)	Accuracy MEAN (%)
**7**	4.5	4.6	4.8	0.2	**3.4**	2.6	**27.2**
	3.1	4.7				52.3	
	3.9	5.0				26.6	
**3**	2.0	1.8	1.8	0.3	**14.5**	12.0	**12.2**
	1.8	1.5				18.6	
	1.9	2.0				6.2	

## Abbreviations

BRCA: Breast Related Cancer Antigens
CV: coefficient of variation
LOB: limit of blank
LOD: limit of detection
MALDI: matrix assisted laser desorption ionization
MS: mass spectrometry
MSI: mass spectrometry imaging
NPs: nanoparticles
PARPi: poly (ADP-ribose) polymerase inhibitors
P-gp: P-glycoprotein
Pt: platinum
QC: quality control
ROI: region of interest
SD: standard deviation
SE: standard error
