# Supplementary material for: Quantitative determination of niraparib and olaparib tumor distribution by mass spectrometry imaging
Source: Int J Biol Sci. 2020 Feb 21;16(8):1363–75. doi: 10.7150/ijbs.41395 (PMC7085221; doi:10.7150/ijbs.41395)
Supplement: Supplementary file 1 — Supplementary figures and tables. [file ijbsv16p1363s1.pdf]

Quantitative determination of niraparib and olaparib tumor distribution by mass spectrometry imaging

Lavinia Morosi[1*], Cristina Matteo[1*], Tommaso Ceruti [1], Silvia Giordano[2], Marianna Ponzo[1], Roberta Frapolli[1], Massimo Zucchetti [1], Enrico Davoli[2], Maurizio D'Incalci[1§], and Paolo Ubezio[1§].

[1] Istituto di Ricerche Farmacologiche Mario Negri IRCCS, Department of Oncology

[2] Istituto di Ricerche Farmacologiche Mario Negri IRCCS, Laboratory of Mass Spectrometry

* L.M. and C.M. contributed equally

§ M.D. and P.U. co-last authors

Corresponding author: Lavinia Morosi, Istituto di Ricerche Farmacologiche Mario Negri IRCCS, via M. Negri 2, 20156 Milano

Phone: +39 02 39014726

Fax: +39 02 39014734

Email: lavinia.morosi@marionegri.it

SUPPLEMENTARY MATERIAL

Supplementary Material and Methods

AuTiO$_2$NPs synthesis

The nanoparticles in use have a Au/TiO$_2$ "nanocomposite" structure, where the TiO$_2$ nanoparticle, with size of few tens of nm, supports onto its surface, smaller Au clusters having size as small as few nanometers. Such nanocomposite particles were produced, in form of a dry powder, employing a commercial system based on Flame Spray Pyrolysis (NPS-20, ParteQ, Germany). The NPS-20 is a bench top system allowing the development and production of tailored nanopowders for R&D purposes. The pyrolysis reactions take place in a burner. The system operation is shortly reported in the following. A liquid solution containing the metal organometallic precursors (details below) was injected, by a syringe pump through a stainless-steel capillary at 3 ml/min, into a nozzle where it was dispersed by an oxygen flow of 3 dm3/min\u0002. The concentric flamelet ring was fed with a mixture of methane/oxygen (CH4 1.5 dm3/min\u0002, O2 3.2 dm3/min\u0002). Additional oxygen (5 dm3/min\u0002) was supplied by the outer sheath flow to assure the complete conversion of the precursors. The powder was collected on a glassfiber filter (GF/A Whatman, Kent, United Kingdom), 150 mm in diameter, placed in a water-cooled holder 400 mm above the nozzle. The solution of nanoparticle precursors was prepared by dissolving Chloroauric acid (HAuCl4 · xH2O, Sigma Aldrich) into acetonitrile and then placing a suitable quantity of the obtained solution into a premixed solution of Titanium (IV) tetra-isopropoxide (Sigma Aldrich) and xylene (Carlo Erba) resulting a final concentration of 0,68 moles/liter of Ti and 0.5% wt Au/TiO2.

MSI data processing pipeline

Raw data of the MS spectra were extracted from the MS acquisition file using a home-made Matlab routine focusing in the mass ranges including the peaks of interest (in our case those of the drugs NIR or OLAP and their deuterated internal standard). Data preprocessing was performed using algorithm described before[1]. For this work a dedicated Excel template was developed in order to face specific issues of different drugs and matrices.

In the main worksheet of the developed template, each column correspond to one specific pixel, whose coordinates (i,j) in the image have been determined on the basis of the information retrieved from the header of the MS file. Each row of the spreadsheet instead contains the signals detected for a given m/z value in that pixel. The user selects a m/z range were the internal standard is expected and the width of the peaks, then the program finds the maximum signal in that range and the corresponding m/z position for each pixel, and it calculates the area ($S_{IntSt}(i,j)$) and the mean m/z value (M_{IntSt} (i,j)) of the internal standard peak. The expected m/z position of the drug peak for that pixel is calculated as $Mexp_{drug}(i,j) = M_{IntSt}(i,j) - \Delta$ where Δ is the known difference between the m/z of the drug and its internal standard. Then a range is selected around $Mexp_{drug}(i,j)$ where the row signals are integrated giving the drug-associated area (A_{drug} (i,j)). Notice that an unknown fraction of $A_{drug}(i,j)$ is background, unrelated to the drug content.

To correct the different efficiencies of ion extraction in each pixel, A_{drug} (i,j) is normalized to the internal standard signal, in the assumption of uniform delivery of the internal standard itself, giving the drug-related signal: $S_{drug}(i,j) = A_{drug}(i,j) / S_{IntSt}(i,j)$.

The excel workbook includes a worksheet (named "distr") with squares cells where a 2D image is generated for any selected row of the main worksheet, enabling in particular to collect $S_{drug}(i,j)$ in a

matrix and to generate an image via a visual basic routine applying a color to the cell (i,j) on the basis of $S_{drug}(i,j)$ and a color scale specified by the user.

Another worksheet (named "distrmed") automatically applies a 3x3 median filter to the image matrix in the worksheet "distr" and generates a filtered image. In this way $S_{drug}(i,j)$ is converted to the corresponding $S^*_{drug}(i,j)$ matrix.

Other worksheets are included in the workbook, enabling the user to generate mask matrices selecting ROIs on the image generated in "distr" or "distrmed" worksheets. This in particular enables to select a mask associated to the tissue, identifying the slice. For this purpose an ion signal from a representative tissue component, have to be identified and extracted in order to distinguish the tissue from the plate to analyze the drug distribution only in "significant" pixels/spectra. We empirically found an ion signal (m/z 393.2) always present in the tumour tissue and absent outside the section that can be used as a marker of tissue presence.

Suitable ROIs are generated to analyze the spots in a scale of known drug standards and to build the calibration curve to convert the $S^*_{drug}(i,j)$ into the final quantitative image $D_{drug}(i,j)$ of the drug concentration (e.g. in terms of pmol/mm^2) measured in each pixel. The calibration curve has usually an intercept higher than zero, accounting for the background non-specific signal. Alternatively, a background subtraction could be made in each pixel, but no tested method was immune from producing artifacts in the present case, particularly in the low concentration range where NIR and OLAP were detected in the present study. For this reason pixel-by-pixel background subtraction was not pursued in this study, and the direct use of the calibration curve, together with the determination of the LOB and LOD was adopted as a more robust procedure. In fact, in order to interpret correctly the image as representative of the (relative) drug distribution it is necessary to specify the limits of blank (LOB) and detection (LOD) levels. Following Armbruster DA et al [2], the LOB was defined as the signal level corresponding to the 95 percentile of repetitions of blank measures and the LOD was identified by the minimum analyte level for which 95% of the repetitions were above the LOB, giving 5% probability to both α and β errors. In our case the LOB was established with tumor samples of untreated animals. The frequency distribution of the normalized drug-related signals in the pixel of these samples, typically including thousands pixels, is representative of the distribution of the non specific contribution of signal, and was used to set the LOB, corresponding to the 95th percentile of the distribution. In order to set the LOD we made serial dilutions of a mother solution with a known drug concentration. 0.2 µl of each dilution were spotted on a tumor section of an untreated animal ("calibration" slection). Signal intensity inside each spot is quite homogeneous within the spots and even more selecting a ROI inside, without borders, including typically 100 pixels. Drug concentration was assumed constant within these ROIs, thus the pixels inside provided replicate measures of the same drug concentration. This nominal drug concentrations (pmol/mm^2) in the ROIs of each spot were calculated, dividing the overall drug amount (the fraction of the known spotted quantity falling in the ROI was calculated dividing the sum of the drug signals in the ROI to that of the entire spot) by the exact number of pixels in the ROI (converted to mm^2 taking into account the dimensions of each pixel).

Supplementary Results

Matrix selection

For the preliminary screening, the 3 matrices (AuNPs, TiO_2NPs and AuTiO_2NPs) were co-spotted with a fixed concentration (100pmol/µL) of niraparib or olaparib on MALDI plate. Resulting mass spectra are shown in Figures S1-S6.

NIR ionized preferentially in positive ion mode as an adduct with Na^+ or K^+ at m/z 343.1 and m/z 359.1 respectively. The ion signal corresponding to NIR was the base peak of the spectrum and the signal intensity was optimal with all the matrices tested. The highest signal intensity was obtained with AuTiO_2. In negative ion mode, the signal was lower or absent and a high level of background noise was detected. As regard OLAP, using AuNPs or AuTiO_2NPs the ionization happened efficiently in positive ion mode as an adduct with Na^+ or K^+ at m/z 457.1 and m/z 473.1 respectively. Using TiO_2NPs the best ionization was obtained in negative ion mode although the peak corresponding to OLAP was not the base peak and the background noise resulted higher than with the other two tested nano-matrices.

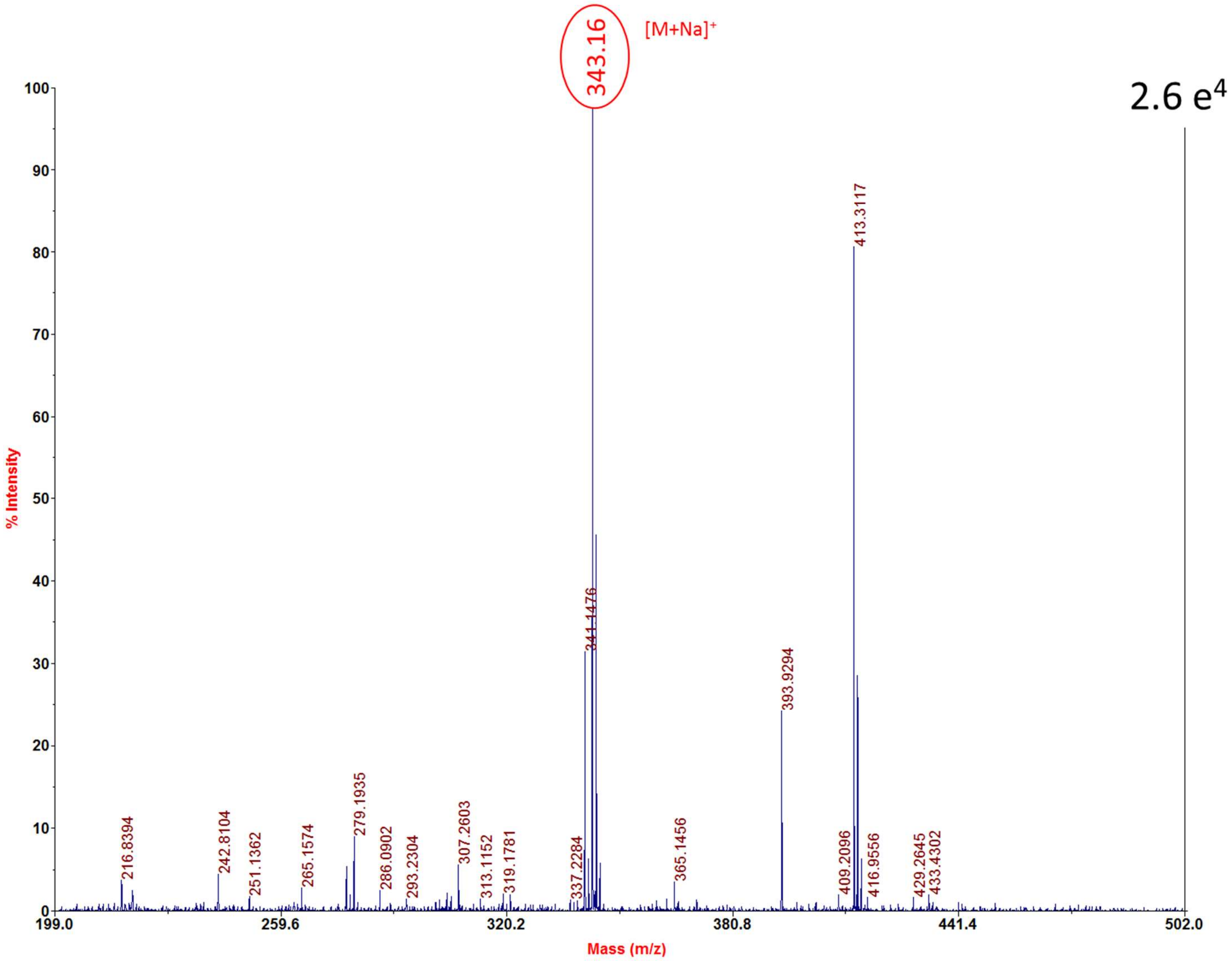

FigureS1: Mass spectra of niraparib (100 pmol/spot) with AuNPs

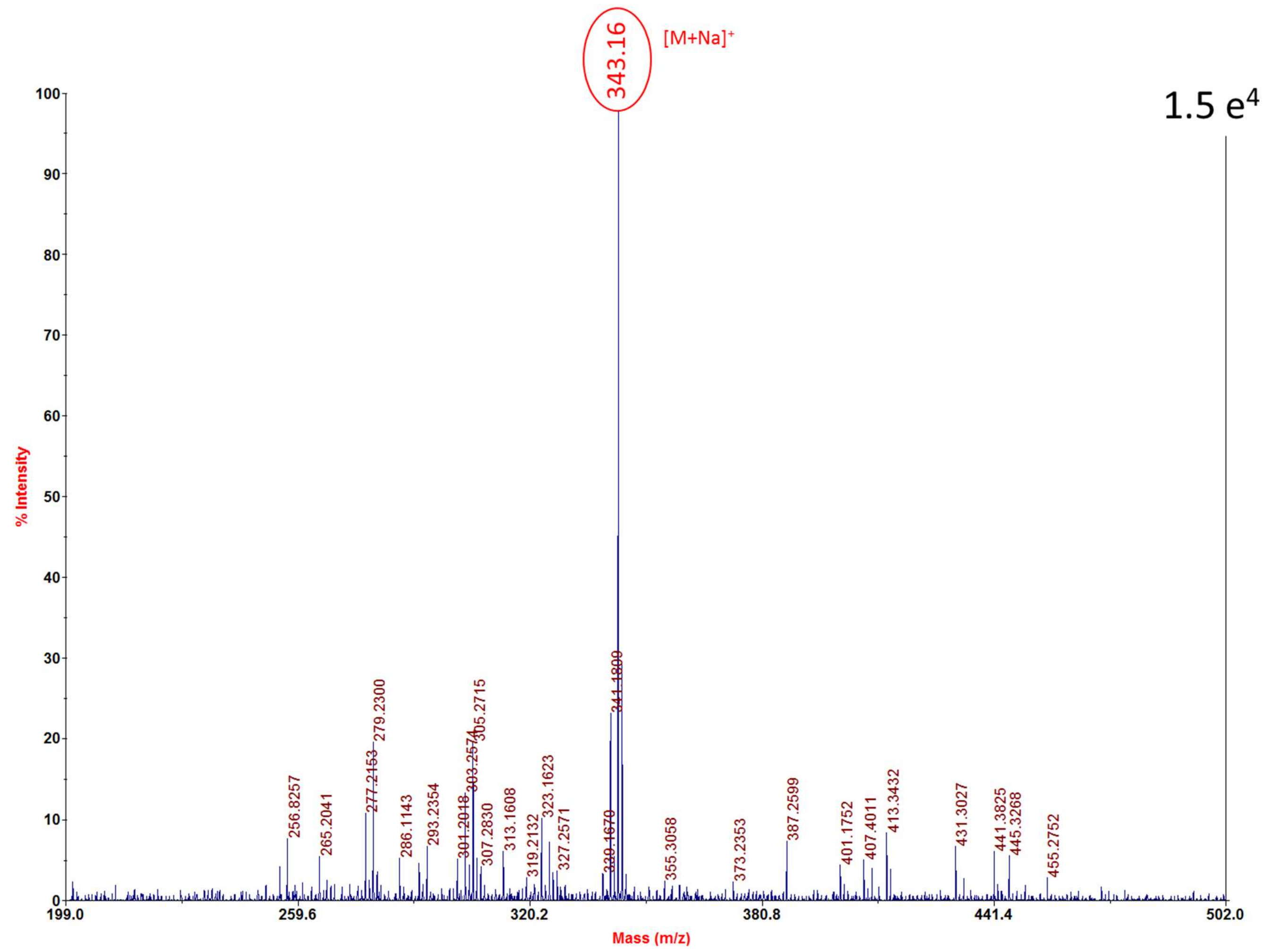

FigureS2: Mass spectra of niraparib (100 pmol/spot) with TiO2NPs

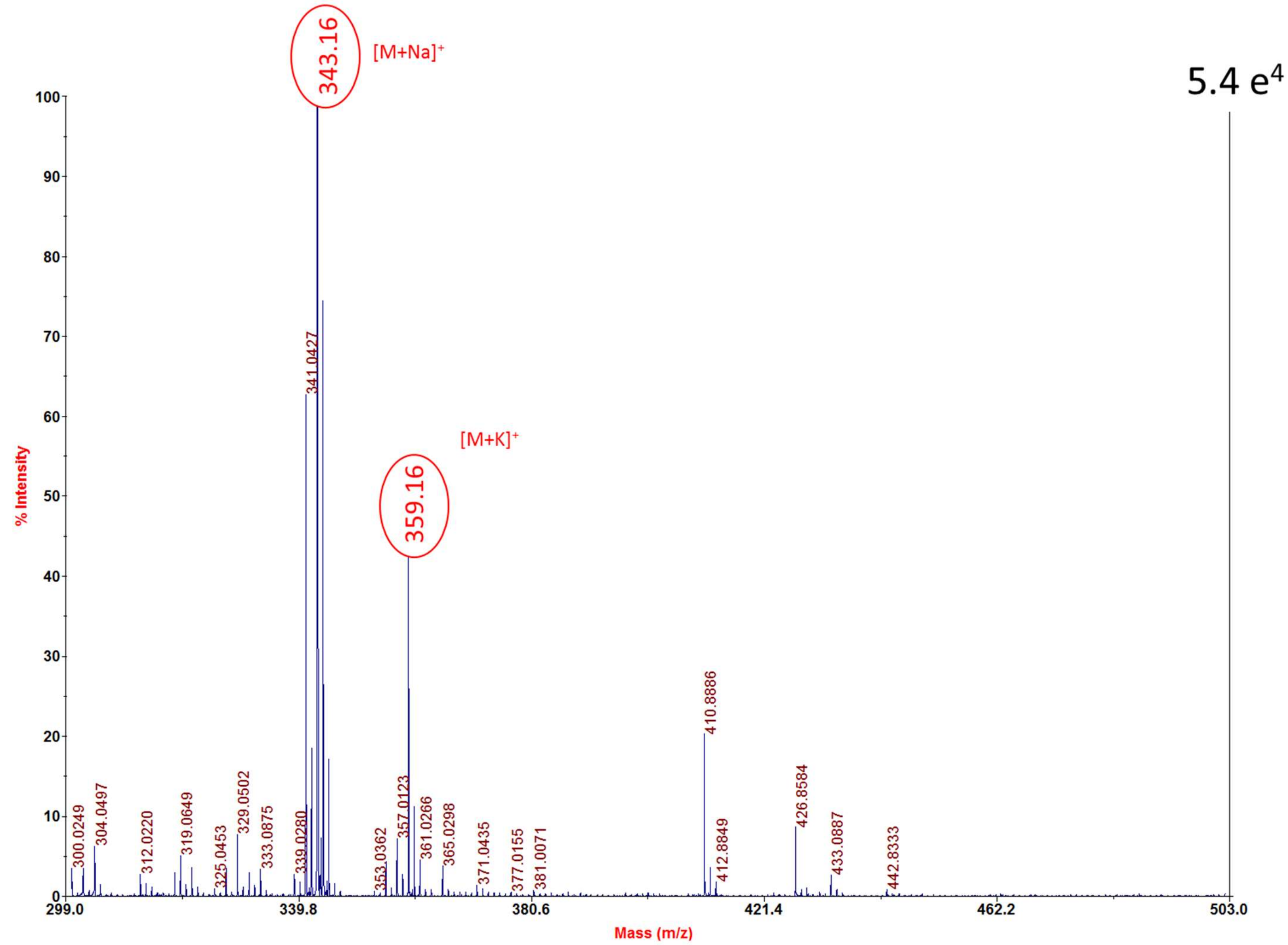

FigureS3: Mass spectra of niraparib (100 pmol/spot) with AuTiO2NPs

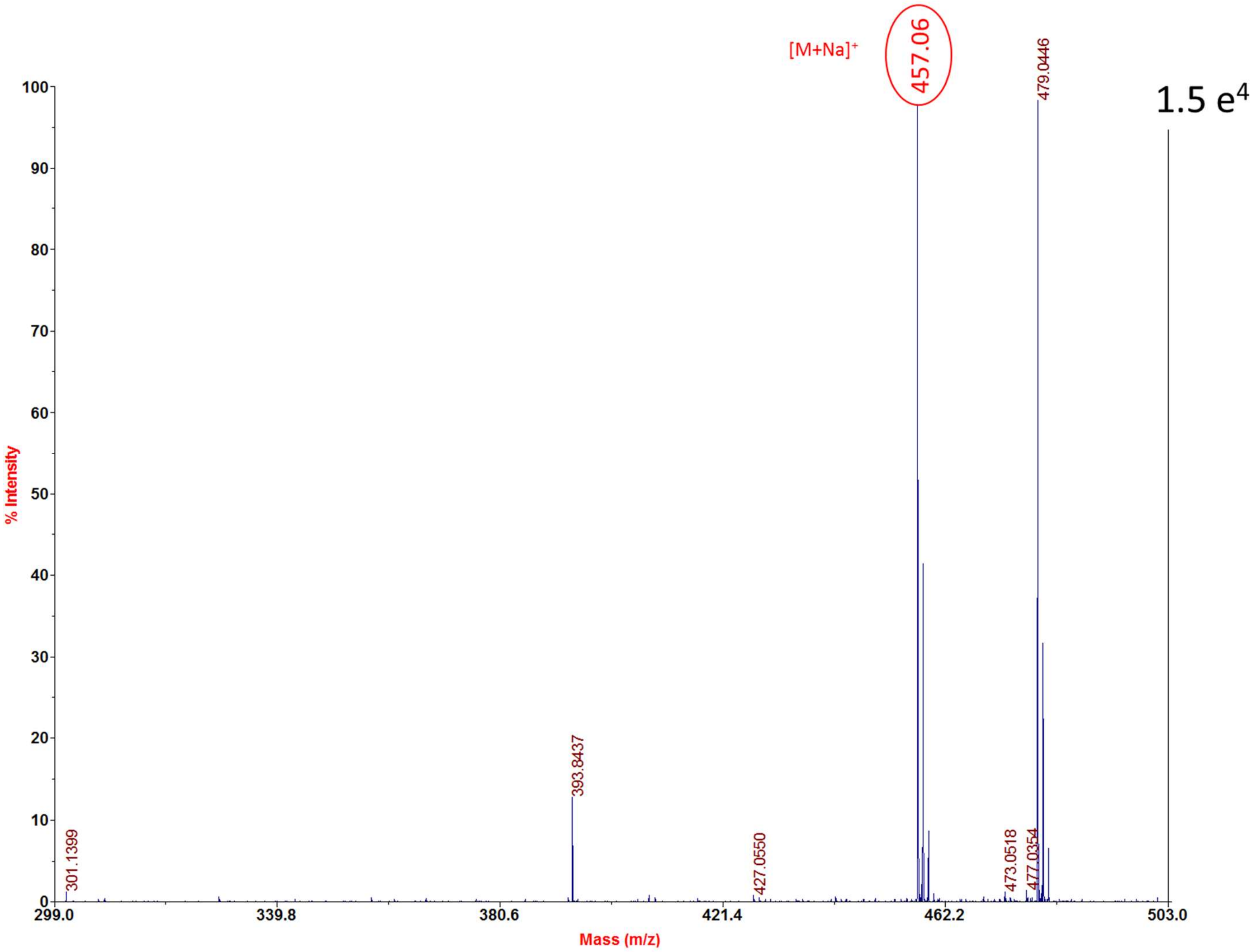

FigureS4: Mass spectra of olaparib (100 pmol/spot) with AuNPs

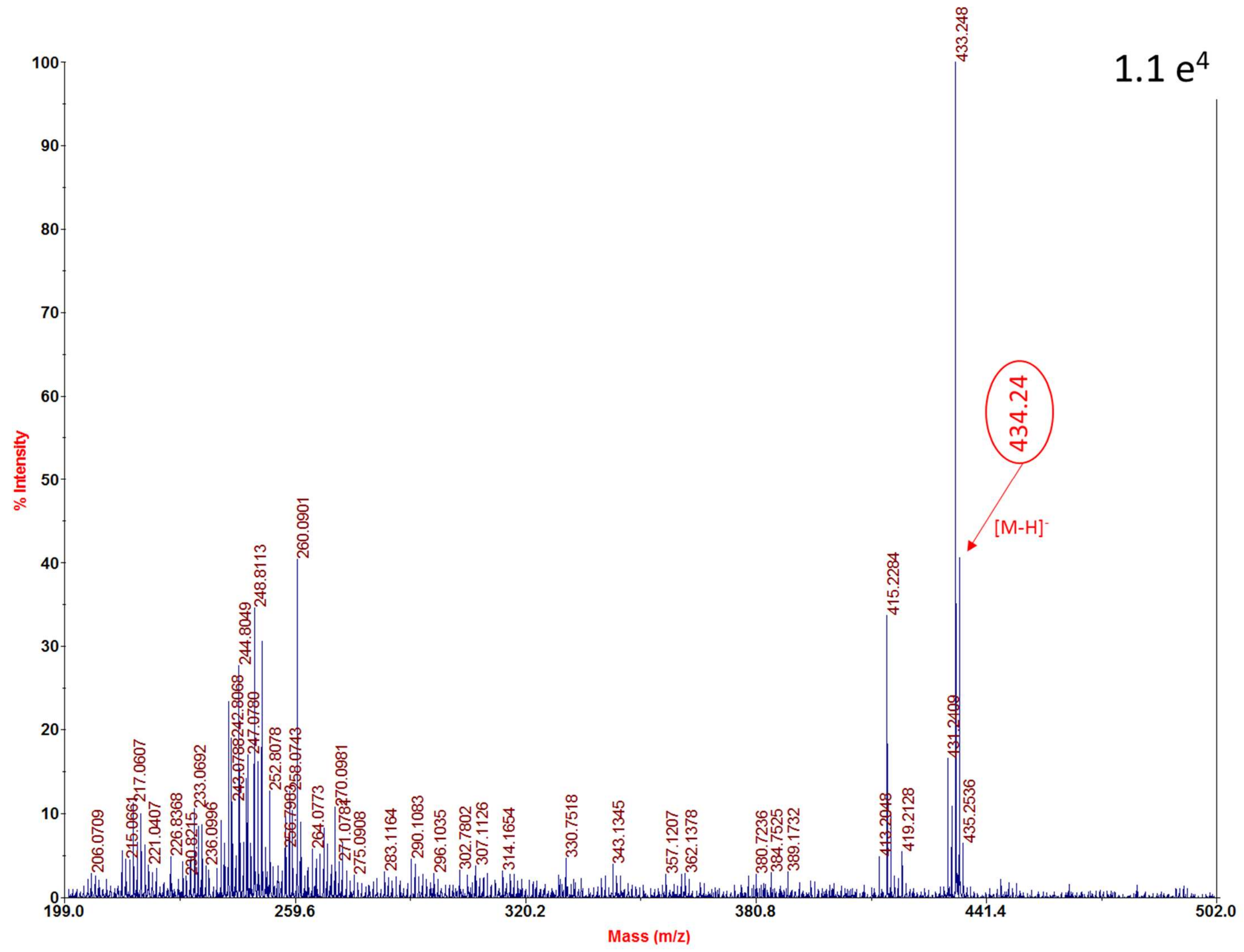

FigureS5: Mass spectra of olaparib (100 pmol/spot) with TiO2NPs

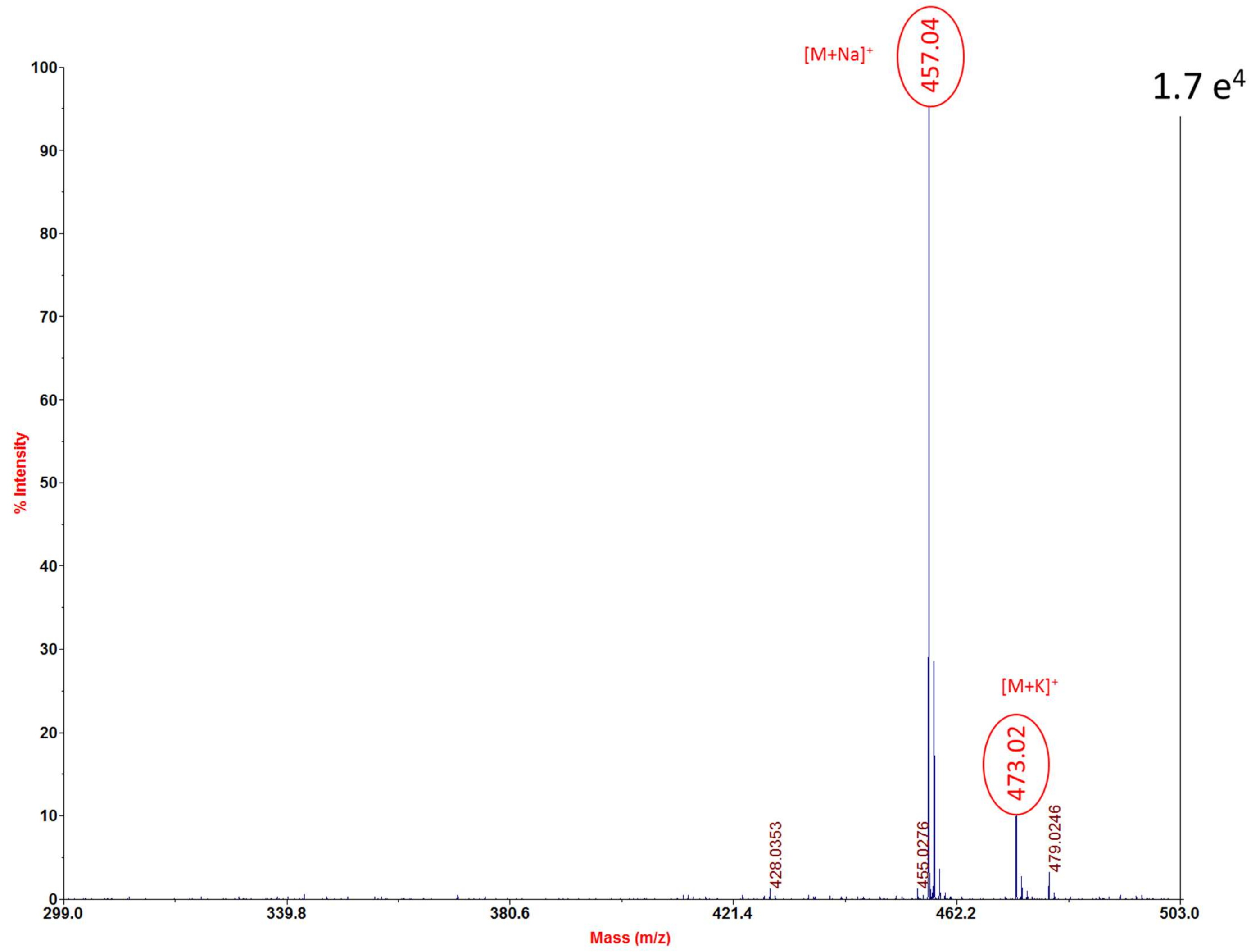

FigureS6: Mass spectra of olaparib (100 pmol/spot) with AuTiO2NPs

Figure S7 depicts the distribution of ion signals of niraparib and olaparib spotted at increasing concentrations on untreated tumor sections with the different nanomatrix. The best result, in terms of lowest detectable concentration, aspecific signals and background noise, was achieved with $AuTiO_2NPs$ for both drugs.

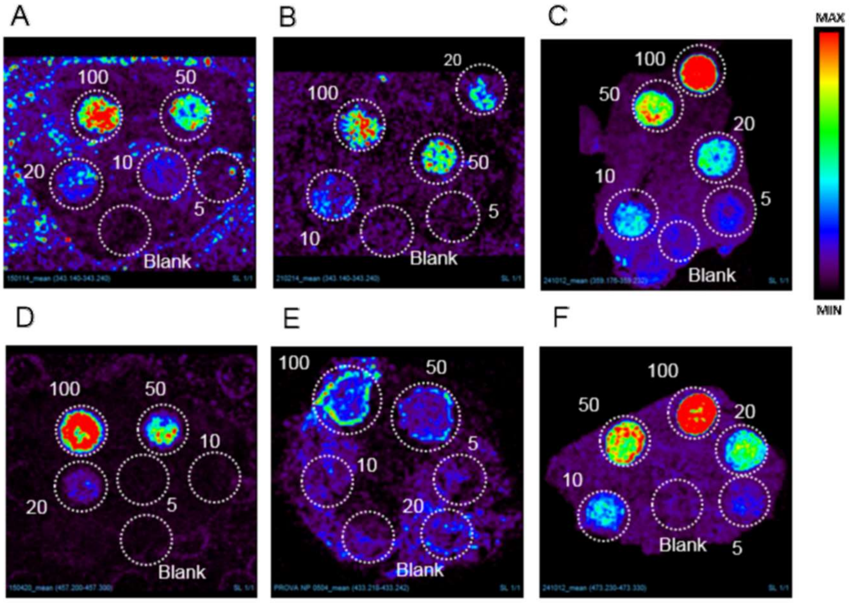

FigureS7: MSI analysis of niraparib (A-B-C) and olaparib (D-E-F) spotted at increasing concentrations on untreated tumor sections with different nanoparticle matrices: AuNPs (A, D), TiO_2NPs (B, E) and $AuTiO_2NPs$ (C, F). Na^+ adduct for niraparib and olaparib with AuNPs, K^+ adduct for niraparib and olaparib with $AuTiO_2NPs$, Na^+ adduct for niraparib and negative ion for olaparib with TiO_2NPs are shown.

Homogeneity of matrix deposition

The homogeneity of matrix deposition was controlled evaluating the variability of the internal standard ion signal comparing different sections (on the same MALDI plate) and different ROI inside the same section (Table S1 and S2). We monitored in addition the variability of an ion signal derived from the nanomatrix (m/z 403.2).

Table S1: Mean ion signal of the deuterated internal standards and of a matrix peak in different tumor sections

section #	m/z 403.2	D8-olaparib	D7-niraparib
1	405.3	284.1	407.6
2	422.2	277.6	409.9
3	380.1	284.1	436.5
4	385.1	275.5	516.5
5	335.9	235.9	422.8
**MEAN**	**385.7**	**271.4**	**438.7**
**SD**	**32.5**	**20.2**	**45.0**
**CV%**	**8.4**	**7.5**	**10.3**

Table S2: Mean ion signal of the deuterated internal standards and of a matrix peak in different ROIs in the same tumor section

ROI #	m/z 403.2	D8-olaparib	D7-niraparib
1	424.2	260.2	414.8
2	427.4	261.8	428.7
3	468.5	299.9	442.6
4	424.4	284.7	419.8
5	401.9	272.9	435.2
**MEAN**	**429.3**	**275.9**	**428.2**
**SD**	**24.2**	**16.6**	**11.3**
**CV%**	**5.6**	**6.0**	**2.6**

Inter-day repeatability

Figure S8 shows the inter-day repeatability of the niraparib and olaparib calibration scales and their weighted linear fitting. The fitting was acceptable in all the calibration curves, with standardized residuals randomly distributed over and below zero..

For niraparib the best-fit slope (m) and y intercept (q) of three independent scales spotted on different working days were similar (m mean±SD: 0.399±0.076, CV%=18.9); q mean±SD: 0.081±0.024, CV%=29.4) while for olaparib the variability of the slope (m mean±SD: 0.150±0.055, CV%=36.7) and y intercept (q mean±SD: 0.057±0.055, CV%=96.8) is higher than in intra-day comparisons.

For niraparib the back-calculated concentration accuracy expressed as the percentage deviation ranged between 4.1% and 16.6% with only the lowest calibration point at 24.0%. Single values are shown in Table S3.

For olaparib the back-calculated concentration accuracy expressed as the percentage deviation was less than 20% for all calibration points except the lowest (37.6%). Single values are shown in Table S4. The inter-day repeatability is not completely assessed and therefore is mandatory to prepare a fresh calibration curve for each plate analyzed to obtain reliable data.

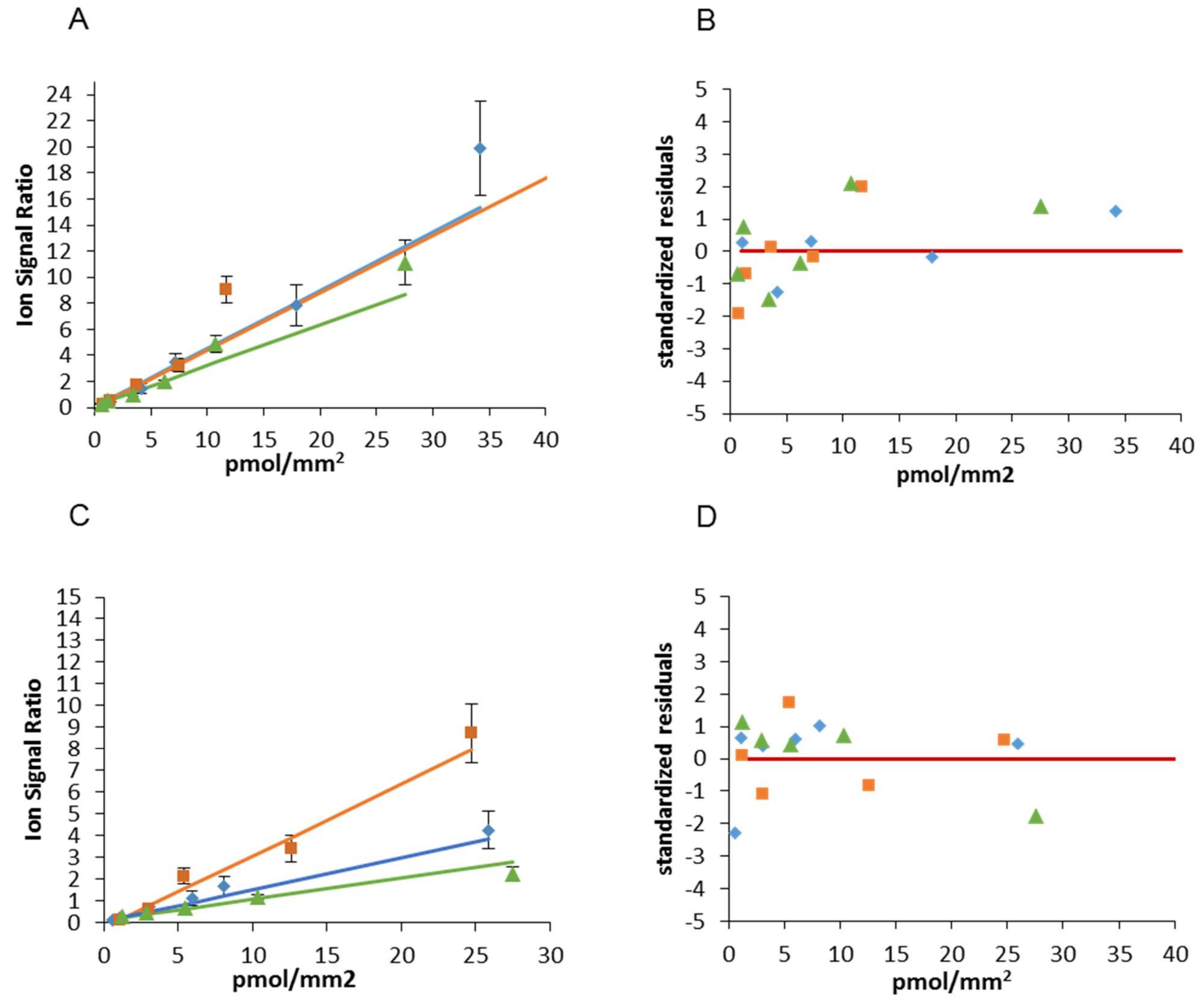

Figure S8: Niraparib (A) and olaparib (C) calibration curves spotted on three different working days and the corresponding standardized residuals (B and D). The red lines indicate the ideal value of the residuals (0).

Table S3: Back-calculated concentration accuracy expressed as the percentage deviation of the different calibration points of three niraparib calibration curves acquired on different working days

Spotted pmol/spot	Nominal pmol/mm^2	Measured pmol/mm^2	Accuracy (%)	Accuracy MEAN (%)
**1**	5.2	5.1	4.2	**24.0**
	4.6	5.7	56.3	
	5.4	6.9	11.6	
**2**	2.1	1.7	2.4	**4.1**
	1.9	2.0	2.8	
	1.9	1.6	7.1	
**5**	1.0	0.7	10.2	**12.9**
	0.6	0.5	1.6	
	0.7	0.7	26.8	
**10**	5.2	5.1	6.3	**8.1**
	4.6	5.7	11.4	
	5.4	6.9	6.6	
**20**	2.1	1.7	38.6	**14.2**
	1.9	2.0	1.0	
	1.9	1.6	3.1	
**50**	1.0	0.7	18.2	**16.6**
	0.6	0.5	13.6	
	0.7	0.7	18.0	

Table S4: Back-calculated concentration accuracy expressed as the percentage deviation of the different calibration points of three olaparib calibration curves acquired on different working days

Spotted pmol/spot	Nominal pmol/mm^2	Measured pmol/mm^2	Accuracy (%)	Accuracy MEAN (%)
**1**	0.6	0.7	21.6	**37.6**
	0.6	0.4	38.4	
	0.7	0.3	52.8	
**2**	1.3	1.1	14.0	**16.2**
	1.2	1.6	33.6	
	1.1	1.1	1.1	
**5**	3.5	2.3	34.9	**19.8**
	2.9	3.2	10.7	
	3.1	2.6	13.9	
**10**	6.1	6.8	11.9	**17.4**
	5.5	5.9	6.9	
	5.4	7.2	33.3	
**20**	11.2	12.2	9.1	**9.9**
	10.4	11.2	8.3	
	12.6	11.1	12.2	
**50**	20.2	25.9	28.0	**18.9**
	27.5	22.2	19.1	
	24.8	27.1	9.4	

Correlation between MSI and LC-MS/MS analysis

A representative LC-MS/MS chromatogram of niraparib at the LLOQ concentration for niraparib and olaparib is shown in figure S9.

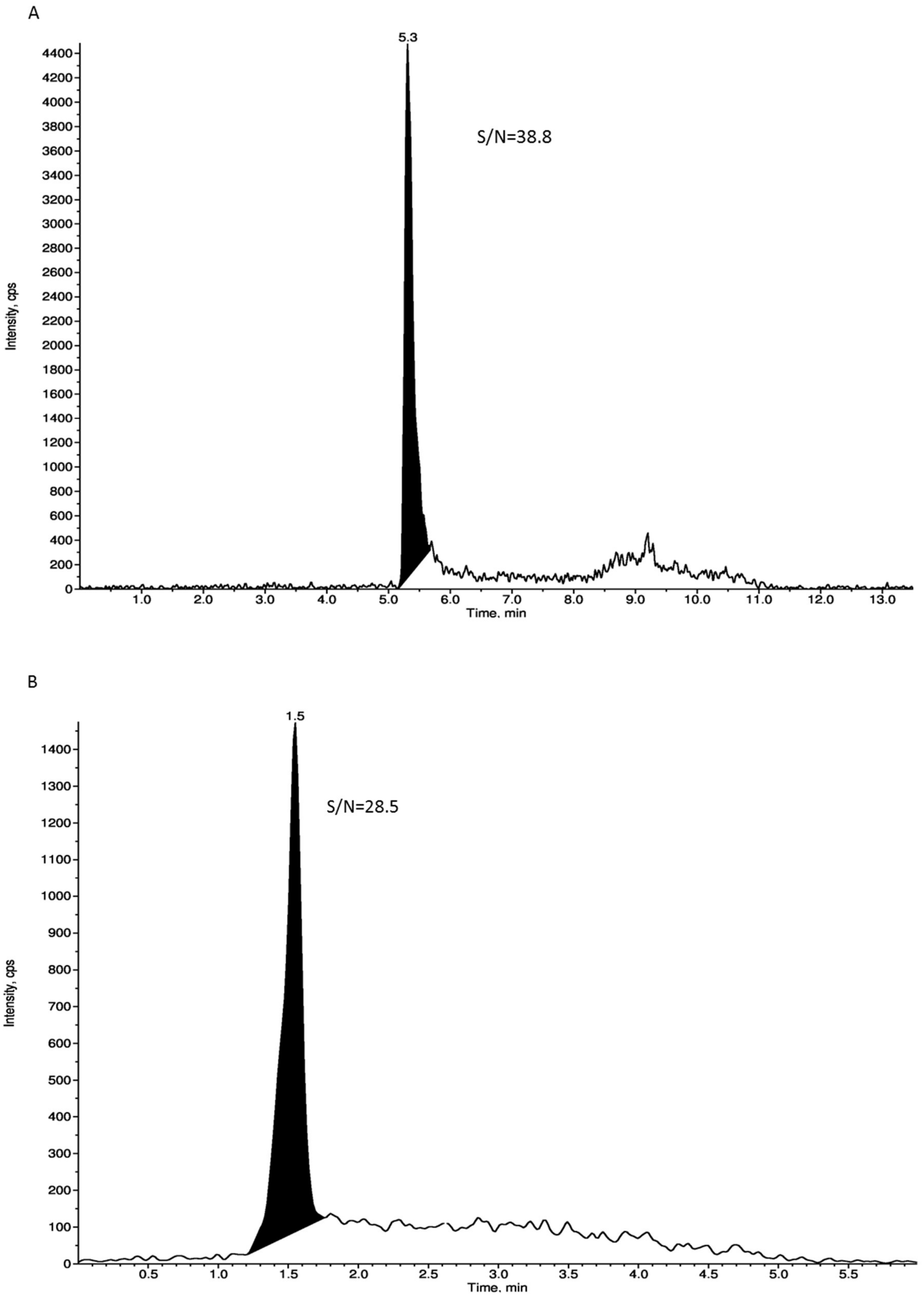

FigureS9: Chromatogram of the quantifier transition at the LLOQ of niraparib (A) and olaparib (B).

The quantitative LC-MS/MS analysis on tumor homogenates confirmed this differential distribution: intratumor concentration of niraparib was found about twice higher in wild type than in A2780/P-gp tumors with both methods(mean: 10.40±1.69 µg/g in A2780*wt* and 5.29±0.55 µg/g in A2780/P-gp (t test p-value=0.00237). The correlation between LC-MS/MS and MSI quantitative results is quite good with an R^2=0.8633 (figure S10). The mean drug concentrations calculated locally in the slices by MSI (mean: 25.74±3.20 µg/g and 9.96±2.93 µg/g in A2780*wt* and A2780/P-gp respectively.) were in the same order of magnitude but higher than the bulk averages measured by LC-MS/MS (Table S5).

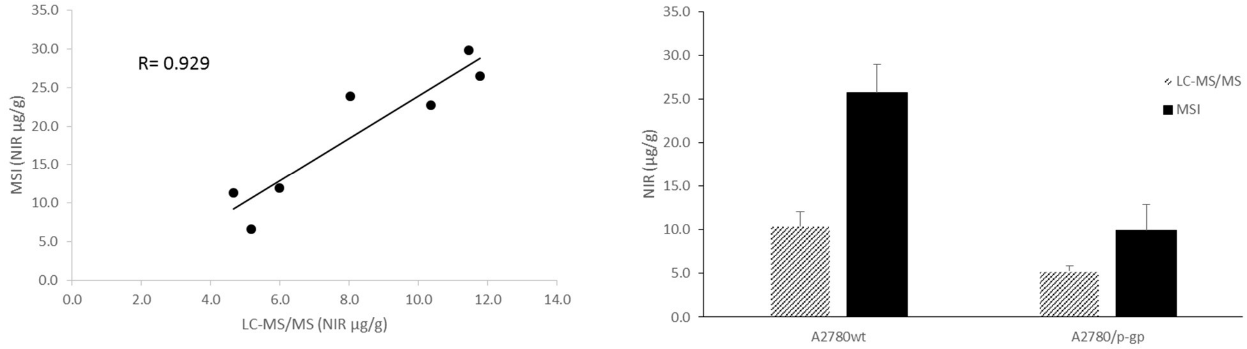

FigureS10: Correlation of niraparib amount in tumor samples by MSI and LC-MS/MS.

TableS5: Niraparib tumor concentration measured by LC-MS/MS compared to MSI

	**LC-MS/MS**			**MSI**		
**Tumor**	**Niraparib (µg/g)**	**MEAN**	**SD**	**Niraparib (µg/g)**	**MEAN**	**SD**
**A2780*wt***	10.36<br>11.44<br>8.04<br>11.77	10.4	1.69	22.67<br>29.88<br>23.88<br>26.52	25.7	3.2
**A2780P-gp**	4.65<br>5.18<br>5.35<br>5.98	5.29	0.55	11.37<br>6.60<br>11.92	10.0	2.9

References:

[1] Falcetta F, Morosi L, Ubezio P, Giordano S, Decio A, Giavazzi R, et al. Past-in-the-Future. Peak detection improves targeted mass spectrometry imaging. Anal Chim Acta 2018;1042:1–10. https://doi.org/10.1016/j.aca.2018.06.067.
[2] Armbruster DA, Pry T. Limit of blank, limit of detection and limit of quantitation. Clin Biochem Rev 2008;29 Suppl 1:S49-52.
